# New loci and candidate genes in spring two-rowed barley detected through meta-analysis of a field trial European network

**DOI:** 10.1007/s00122-025-04934-8

**Published:** 2025-06-23

**Authors:** Francesc Montardit-Tarda, Ana M. Casas, William T. B. Thomas, Florian Schnaithmann, Rajiv Sharma, Salar Shaaf, Chiara Campoli, Joanne Russell, Luke Ramsay, Micha M. Bayer, Stefano Delbono, Marko Jääskeläinen, Maitry Paul, Frederick L. Stoddard, Andrea Visioni, Andrew J. Flavell, Klaus Pillen, Benjamin Kilian, Andreas Graner, Laura Rossini, Robbie Waugh, Luigi Cattivelli, Alan H. Schulman, Alessandro Tondelli, Ernesto Igartua

**Affiliations:** 1 Estación Experimental de Aula Dei–Consejo Superior de Investigaciones Científicas (EEAD-CSIC), Avenida Montañana 1005, 500059 Saragossa, Spain; 2 James Hutton Institute, Errol Road, Invergowrie, Dundee, DD2 5DA UK; 3 Martin-Luther-University Halle-Wittenberg, Betty-Heimann-Str. 3, 06120 Halle (Saale), Germany; 4 Leibniz Institute of Plant Genetics and Crop Plant Research (IPK), Corrensstrasse 3, 06466 Gatersleben, Germany; 5 Scotland’s Rural College, Peter Wilson Building, The King’s Buildings, West Mains Road, Edinburgh, EH9 3JG UK; 6 Università Degli Studi Di Milano, Via Celoria 2, 20133 Milan, Italy; 7 Council for Agricultural Research and Economics (CREA), Research Centre for Genomics and Bioinformatics, Via San Protaso 302, 29017 Fiorenzuola d’Arda, Italy; 8 Institute of Biotechnology, University of Helsinki, 00014 Helsinki, Finland; 9 Viikki Plant Sciences Centre, University of Helsinki, 00014 Helsinki, Finland; 10 Natural Resources Institute Finland (LUKE), 00014 Helsinki, Finland; 11 International Center for Agricultural Research in the Dry Areas (ICARDA), 10100 Rabat, Morocco; 12 Dundee University at SCRI, Invergowrie, Dundee, DD2 5DA UK; 13 Global Crop Diversity Trust, Platz Der Vereinten Nationen 7, 53113 Bonn, Germany

## Abstract

**Key message:**

A dense genome-wide meta-analysis provides new QTLs, reveals breeding history trends and identifies new candidate genes for yield, plant height, grain weight, and heading time of spring barley.

**Abstract:**

This study contributes new knowledge on quantitative trait loci (QTLs) and candidate genes for adaptive traits and yield in two-rowed spring barley. A meta-analysis of a network of field trials, varying in latitude and sowing date, with 151 cultivars across several European countries, increased QTL detection power compared to single-trial analyses. The traits analysed were heading date (HD), plant height (PH), thousand-grain weight (TGW), and grain yield (GY). Breaking down the analysis by the main genotype-by-environment trends revealed QTLs and candidate genes specific to conditions like sowing date and latitude. A historical look on the evolution of QTL frequencies revealed that early selection focused on PH and TGW, likely due to their high heritability. GY selection occurred later, facilitated by reduced variance in other traits. The study observed that favourable alleles for plant height were often fixed before those for grain yield and TGW. Some regions showed linkage in repulsion, suggesting targets for future breeding. Several candidate genes were identified, including known genes and new candidates based on orthology with rice. Remarkably, the *deficiens* allele of gene *Vrs1* appears associated with higher GY. These findings provide valuable insights for barley breeders aiming to improve yield and other agronomic traits.

**Supplementary Information:**

The online version contains supplementary material available at 10.1007/s00122-025-04934-8.

## Introduction

Barley (*Hordeum vulgare* L.) is the fourth-ranking cereal in the world, and one of the most important crops in Europe, in terms of cultivation area and economic relevance (Dawson et al. 2015; Looseley et al. 2020). In Europe, barley has been the subject of intensive breeding for over 100 years. Competitive breeding in the spring two-rowed pool, with thorough use of the traditional “cross the best with the best and hope for the best” strategy, has increased concerns about possible genetic erosion in the cultivated germplasm pool. This process has led to the preferential selection of some genomic regions and to an overall decrease in genetic diversity, particularly in the spring barley pool (Kolodinska Brantestam et al. 2004; Dziurdziak et al. 2022; Schmidt et al. 2023). Indeed, Tondelli et al. (2013) detected signs of extinction of diversity in some genomic regions. Intensive breeding activities usually produce, inadvertently or intentionally, fixation of alleles with large effects on important target traits. However, genetic variation is still present (Tondelli et al. 2013), although finding QTLs, even with small effects, becomes harder. One way to detect minor QTLs is by relying on extensive phenotyping and meta-analysis (Muñoz-Amatriaín et al. 2020). In many European regions, barley with spring growth habit is sown between February and May, to avoid harsh winters. This is mandatory in Nordic countries and other European areas with harsh winters, particularly in Eastern Europe, but cultivation of spring-type barley occurs throughout Europe. Its relevance is increasing for two reasons. On the one hand, increasing winter temperatures allow its cultivation in areas of Europe (like Germany, Italy, Spain, or Switzerland) where winter barley and autumn/winter sowings were prevalent. On the other hand, the main economic boost for barley breeding in Europe has been, and still is, malting quality, a sector largely dominated by spring two-rowed types; consequently, breeding efforts have been particularly intense within this pool, giving rise to malting cultivars as productive as the best feed barleys.

Genome-wide association (GWA) studies have been widely used in barley to find genomic regions of interest for a large variety of agronomic characters (Igartua et al. 2019; Thomas 2020). In some cases, candidate genes were identified and validated, making for straightforward breeding. Even so, this is only possible when large diversity panels are available, combined with enough marker density. Marker density provided by the 50k SNP chip (Bayer et al. 2017) gives the opportunity to search for candidate genes in association studies. In narrow germplasm sets, linkage disequilibrium (LD) should be high; hence, relatively low marker density is sufficient to pinpoint QTL regions in GWA studies (GWAS). However, to differentiate cultivars that are very close and be able to track alleles of candidate genes, higher marker densities eventually are needed. This is likely the case for the cultivated spring two-rowed barley pool. Dense genotyping can be achieved using exome capture data, which is available in barley (Mascher et al. 2013; Russell et al. 2016; Chen et al. 2022). Another aspect that has not been fully exploited in GWA is the information from multi-trial studies. With few exceptions (for instance Bustos-Korts et al. 2019), these are analysed as the mean across all trials, or by identifying the intersection of associated markers between single-trial analyses. These methods do not make full use of the potential of independent effects tested in multiple sites to detect QTLs (Muñoz-Amatriain et al. 2020).

This study aims at finding new QTLs for relevant agronomic traits in spring two-rowed barley cultivars through the meta-analysis of new genome-wide associations of field trials from two related European collaborative projects, and at indicating new candidate genes that may become new targets for barley breeding in Europe.

## Materials and methods

### Plant material

A collection of 164 spring two-rowed cultivars released in Europe during the 20^th^ century was tested in the framework of two barley European funded projects where most of the co-authors collaborated: EXBARDIV (http://pgrc.ipk-gatersleben.de/barleynet/projects_exbardiv.php) and CLIMBAR (https://project-wheel.faccejpi.net/climbar/). The two projects included extensive sets of European genotypes, described in Tondelli et al (2013), but only spring two-rowed cultivars common to both projects were kept for this study. Spring two-rowed barleys were selected due to their economic importance in Europe. A principal component analysis for marker data was carried out with the *SNPRelate* package (Zheng et al. 2012) in R (R Core Team 2024). Cultivars clearly outside the principal cloud of points were filtered out (Fig. S1). Discarded cultivars either originated in southern Europe (likely representing distinct germplasm pools) or had introgressions from exotic parents. A total of 151 cultivars that did not show a clear population structure were kept for further analyses (Table S1).

### Phenotypic evaluation, curation and analysis

Field trials were carried out in 2009 and 2010 within the EXBARDIV project, and in 2016 and 2017 in the framework of the CLIMBAR project, in the United Kingdom (UK), Finland, Germany, Italy, Spain, and Morocco (Table 1, Table S2). All trials consisted of plots of four to eight rows, 2–3 m long, and 1–1.5 m wide, in two replicates, following alpha-lattice designs, with plots managed according to local practices for sowing rate and chemical plant protection. The 151 selected cultivars were tested across all trials. Flowering time (HD, days from sowing to appearance of the spike out of the flag leaf sheath, Z55, according to Zadoks et al. 1974), plant height (PH, cm, length from the ground to the tip of the spike, without awns, average of five plants), grain yield after combine harvest (GY, t ha^−1^), and thousand-grain weight (TGW, g) were recorded. Raw phenotypic data from EXBARDIV were retrieved for the 2009 and 2010 seasons, partially reported in Xu et al. (2018). Plant height data of 2016 and 2017 trials were analysed, using a different approach, in Bretani et al. (2022). Phenotypic data were curated for outlier data. Two trials (MAR17 and DE2-10) were fully discarded due either to consistently low correlation coefficients with the rest (Fig. S2) or to low overall data quality. Data from 16 trials were kept for HD, PH and GY, while TGW was recorded from 15 trials only. Broad-sense heritability (H^2^) on an entry-mean basis was calculated per trait.

Best linear unbiased estimators (BLUEs) were calculated with Genstat 20 (VSN International 2022). In each trial, the best spatial correction model was used, with the simplest model being a randomized complete block design; the full model including replicates, autoregressive order 1 in rows and columns, and additional contributions from significant random row and column factors (Table S2). Chi-square tests were performed for models differing by a single factor. The most parsimonious model for each trait was chosen, the last in which the inclusion of a spatial correction factor improved the model significantly. If a given cultivar had missing data in three or less trials due to outlier curation or field-trial agronomic issues, i.e. pests, its phenotype was imputed with the value corresponding to the percentile of that trait for the missing cultivar in the average of the remaining trials. In total, 21, 21, 27 and 17 values were imputed for HD, PH, GY, and TGW, respectively, representing less than 0.1% per trait.

An additive main effects and multiplicative interaction analysis (AMMI) was done for each trait with Genstat 20 (VSN International 2022). These analyses were used to cluster the trials into mega-environments, following the main direction of genotype-by-environment interaction (GEI) per trait.

### Genotyping

The lines were genotyped with a 50k Illumina Infinium SNP Array (Bayer et al. 2017). Missing data were imputed with Beagle 5.0 (Browning et al. 2018), as described in Bretani et al. (2022). After imputation, 40,639 markers remained. For further analysis, markers with a minor allele frequency equal to or higher than 0.05 were kept (28,988 markers). Physical positions of markers were retrieved from both MorexV1 (Mascher et al. 2017) and MorexV3 (Mascher et al. 2021) genome sequences. Additional genotyping for flowering time genes and *Vrs1* (main gene determining spike type) was performed for all lines, with specific markers developed as described in Table S1.

### Genome-wide association study and meta-analysis

Association analyses at the single-trial level were carried out for all phenotypes and trials using a mixed linear model (MLM) implemented in the GAPIT package (Wang and Zhang 2021) in R (R Core Team 2024), with a genomic kinship matrix for adjustment of relatedness, calculated with a randomly selected set of 10% of markers (Fig. S3). A Bonferroni multiple test threshold was used to detect single-trial marker–trait associations (MTA), calculated as the logarithm of 0.05 *P*-value divided by the number of markers (− log_10_(*P*-value) = 5.76). A more liberal threshold (− log_10_(*P*-value) = 4) was applied to identified suggestive associations.

The results of a single-trial GWA per trait were meta-analysed with the software METAL (Willer et al. 2010), using the sample size strategy, for the whole set of trials, and for the best grouping of trials indicated by the AMMI analysis. For grain yield, ITA16 and ITA17 were discarded for meta-analysis due to the high dispersion within each trial. A meta-GWA threshold was calculated as the minimum *P*-value detected by 1,000 meta-analyses of 1,000 permutations per trial, for each phenotype and combination of trials. Markers with a higher − log_10_(*P*-value) than the threshold were declared as a marker–trait association (MTA). Neighbouring MTAs were grouped into single QTL with two different criteria. First, MTAs from the same chromosome were grouped according to a cluster analysis, as reported in Looseley et al. (2020). The marker with the largest association per QTL was declared as a flag marker. Then, flag markers from the same chromosome, which were in the same LD block (detailed below), were merged.

### Linkage disequilibrium analysis

A basal genomic LD threshold was computed. This threshold was estimated as the square of the 95^th^ percentile of the distribution of unlinked r^2^ values (square root transformed), as in Breseghello and Sorrells (2006). This distribution was fitted with the values of the interchromosomal r^2^ between 200 random markers per chromosome, discounting the population structure using the r2v parameter, using the R package *LDcorSV* (Mangin et al. 2012), which considers kinship relatedness. For each chromosome, intrachromosomal LD block size was calculated using r2v, for pairwise LD values between 500 random markers per chromosome. Chromosomal LD decay was calculated as the point where a loess regression intercepted with the basal genomic LD, using R package *fitdistrplus* (Delignette-Muller and Dutang 2015); this procedure served to merge the flag markers of several MTAs into a single QTL.

To search for candidate genes, the confidence region for each QTL was calculated. Local LD decay around each flag marker was estimated fitting a loess regression to the pairwise LD values from the closest 400 markers. The confidence region was defined as the distance from the flag marker to the point where the loess curve decreased to the basal genomic LD threshold. When the loess curves did not converge, the chromosomal LD was used instead to declare confidence intervals.

### Meta-analysis multilocus model

The markers found in the meta-analysis may still present some multicollinearity. To reduce it, sequential multivariate analyses of variance were carried out for each trait, with R package *car* (Fox and Weisberg 2019). The flag markers were introduced as independent variables, and the genotypic values for each trial were the dependent variables. Markers were introduced sequentially at each step, keeping in the model the most significant one at each round. The final model summarizes the set of markers most likely having a combined independent effect for all trials, on each trait.

### GWA enrichment with exome capture markers

Markers from exome capture (EC) sequencing were used for the refinement of peak regions found with the 50k SNP chip markers. Read mapping, marker discovery and quality control (QC) of the EC data was carried out as described in Chen et al. (2022). Filtering of variants was carried out following the same principles as in Chen et al. (2022), but using a minor genotype frequency of 5% in order to produce a smaller callset with more robust markers. Samples without exome capture data or with failed QC were imputed using the 50k markers as a backbone. For each QTL, all available markers (50k + EC markers) within its local LD block were retrieved and a single-trial GWA and its meta-analysis were run again. Exome capture markers with higher − log_10_(*P*-value) than that of the flag marker were considered indicators of a possible candidate gene. Relevant annotations of homologs from other species and gene expression (Milne et al. 2021; Li et al. 2023) in tissues related to the phenotype were considered as additional pointers for possible candidate genes.

Homologues of the candidate genes from *Arabidopsis thaliana*, rice, and wheat were identified with protein BLAST in order to gather functional information using Ensembl Plants (Yates et al. 2022). Only the top homologue with an identity above 80% was considered. Orthologues of *Oryza sativa* subsp. *japonica* of each candidate gene were identified with Ensembl Plants. FunRiceGenes (Huang et al. 2022) was used to check whether a rice orthologue was a trait-related gene.

### Allele frequency shifts over time of cultivar release

The genotypic panel used is representative of the progression of spring barley breeding in Europe. The wide range in the cultivars’ year of release enables tracking of the fate of QTLs in parallel with the history of European spring barley breeding. The cultivars were grouped by year of release into four groups, with the number of cultivars in parentheses: 1920–1959 (n = 18), 1960–1979 (38), 1980–1999 (69) and 2000- (29), respectively. Mean allele frequencies in 250 rolling windows per chromosome were calculated for the four groups of cultivars. MetaQTLs allele frequencies were also calculated for the same four classes, to describe any trends likely due to breeding. Allele frequency shifts of metaQTLs, and genome-wide rolling windows, were determined as the difference between the allele frequencies of the oldest and most recent group of cultivars.

## Results

### Field-trial performance and genotype-by-environment interactions

The field trials represented a varied range of latitudes, climates, and edaphic and agronomic conditions. Accordingly, grain yields were highly variable, between 2.8 and 8.0 t ha^−1^, with an overall yield of 4.9 t ha^−1^. Autumn- or winter-sown trials in Spain and Morocco showed the lowest yields, while autumn-sown trials in Italy and spring-sown trials at northern latitudes were more productive (Table 1, Fig. S4). The largest variation in days from sowing to heading was mainly caused by sowing date. The trials that were autumn-sown in Italy or Spain, as well as the winter-sown trial in Morocco, experienced longer cycles, followed by the late-winter-sown trials in Italy (ITA09 and ITA10, sown in February and early March), and by all the spring-sown trials. Among the latter, Scottish trials showed longer seasons than German and Finnish trials. Within sowing dates, those differences in cycle lengths were probably caused by different rates of accumulation of growing degree days. Plant height also varied widely between trial means, from 52 to 90 cm, suggesting an effect of the different environmental and climatic conditions of each trial. Thousand-grain weight (TGW) varied between 33 g, for some southern locations, to 51 g in the Scottish trials, indicating highly variable grain filling conditions. Grain yield trial means showed a tight correlation of 0.84 with plant height (a surrogate of biomass) and a moderate correlation with TGW (0.60), indicating the importance of the biomass formation phase, throughout the entire season, and of the conditions prevalent during grain filling for grain yield build-up (Fig. S5).

**Table 1:** Field-trial network; locations and years. Phenotype means, standard deviation, and broad sense heritability

			Heading time (days after sowing)		Plant height (cm)		Grain yield (t/ha)		Thousand-grain weight (g)	
Country	Trial code	Sowing date	Mean	SD	Mean	SD	Mean	SD	Mean	SD
Morocco	MAR16	06/01/16	104.0	4.5	71.3	7.2	3.25	0.55	NA	NA
Spain	ESP16	11/11/15	163.7	6.6	63	8.8	2.98	0.67	33.2	3.3
Spain	ESP17	15/11/16	154.1	4.6	54.1	6.5	2.85	0.5	39.1	3
Italy	ITA16	05/11/15	167.8	4.0	86.4	6.9	7.98	1.13	38.4	5.2
Italy	ITA17	08/11/16	174.5	4.9	84.3	6.9	6.79	1.03	46.6	4.6
Italy	ITA09	17/02/09	96.3	2.9	65.3	6.1	4.55	0.74	43.6	3.1
Italy	ITA10	01/03/10	93.6	3.8	52.4	5.8	3.14	0.69	42.5	2.6
Germany	DE1-09	31/03/09	62.8	3.3	76.2	7.4	3.91	0.76	35.9	4.6
Germany	DE1-10	06/04/10	67.5	2.2	83.2	8.3	4.74	0.76	46.5	3.7
Germany	DE2-09	03/04/09	66.5	2.9	89.9	12.8	6.55	0.66	50.1	3.4
UK	GBR09	25/03/09	90.7	2.5	72.3	10.8	5.51	0.63	46.1	3.4
UK	GBR10	01/04/10	78.9	2.6	82	9.7	5.24	0.76	46.9	3.9
UK	GBR16	16/03/16	89.5	2.3	89.8	14.2	6.21	0.73	50.4	3.2
UK	GBR17	29/03/17	83.4	2.2	88.1	10.4	7.04	0.79	51.0	3.6
Finland	FIN16	11/05/16	53.3	3.4	49.4	6.3	2.94	0.6	35.8	3.1
Finland	FIN17	19/05/17	53.9	1.9	69.9	7.5	5.43	0.56	49.2	3.6
Heritability (H^2^)			0.822		0.869		0.216		0.836	

**Fig. 1:**
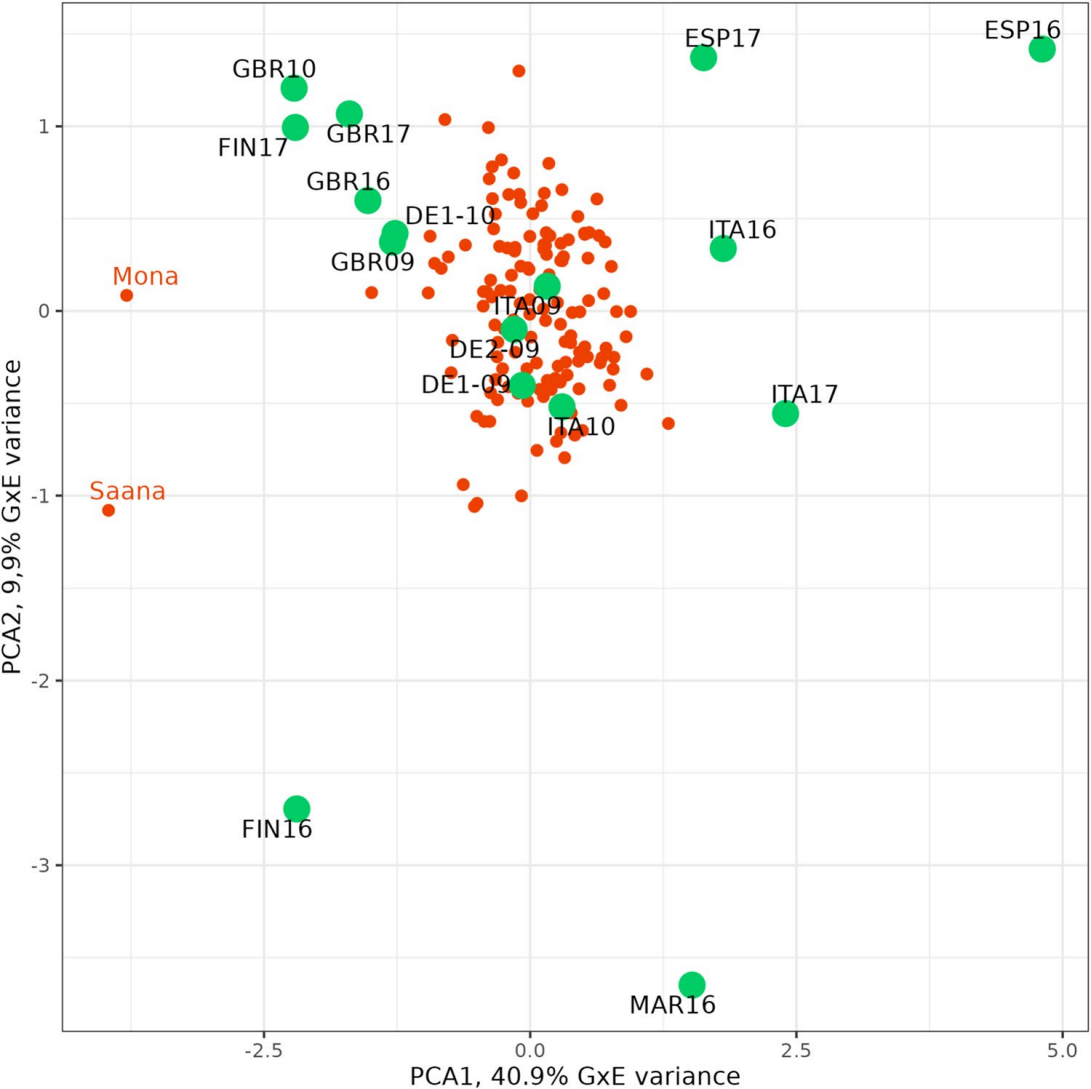
Plot of first two principal components of the AMMI analysis for heading date (Z55) of 151 spring two-rowed cultivars over 16 field trials

For all the traits, both the genotypic and genotype-by-environment (G x E) interaction effects were significant. In general, G x E was more relevant for GY or TGW than for HD or PH but, in all cases, the genotypic sum of squares was much larger. The G x E patterns revealed by the AMMI analysis were different for each trait. For grain yield, most trials formed a tight cloud, except those of ITA16 and ITA17, both being trials with high yields having a large effect on overall G x E variance (Table 2, Fig. S6). For heading date (Fig. 1), the first principal component reflected mainly a difference between sowing dates, with autumn-sown (ESP16, ESP17, ITA16, ITA17) or winter-sown trials (MAR16) showing large positive loadings, spring-sown ones (DE1-09, DE1-10, DE2-09, GBR09, GBR10, GBR16, GBR17, FIN16, FIN17) placed opposite to them, and intermediate-sowing dates (ITA09 and ITA10) in a halfway position. ITA09 and ITA10 were, however, classified as spring trials for meta-analysis. The first principal component explained a large proportion of G x E and was strongly influenced by the differential behaviour of Nordic cultivars Mona and Saana. These cultivars were relatively late in the northernmost environments and very early in the autumn-sown trials. This was also evidenced by the comparison of the differences between the heading date of these cultivars and the overall mean at each trial (Fig. S7). The distribution of trials over the first component for plant height showed a geographic pattern, with most southern environments (Italy, Morocco and Spain) on one side of the first axis and most northern towards the opposite side (Finland, Germany, and the UK). For TGW, the unique behaviour of ITA16 was the main cause of the very large first principal component.

**Table 2:** Multi-environment analysis of variance for BLUEs of four traits. Mean squares are presented (ms). As the replicate layer is not present, the variance ratio (vr) for genotype and environment is calculated with the G x E term in the denominator, becoming a stringent test, as this variance adds true G x E variance to the error variance. G x E variance is broken down in the first four components of an AMMI analysis for each trait, each tested for significance against the residual G x E variance left after removing the variance accounted for by each principal component (PC). The significance of each source of variation is described with asterisks in the vr column: * as 0.05 ≥ *P*-value > 0.01, ** as 0.01 ≥ *P*-value > 0.001, and *** as *P*-value ≤ 0.001.

		Grain yield		Heading date		Plant height		Thousand-grain weight	
Source	df	ms	vr	ms	vr	ms	vr	ms	vr
Genotype (G)	142	3.01	7.9**	117	27.9**	856	34.5**	124.9	22.3**
Environment (E)	15	397.3	1049.9**	249,336	59,620.4**	26,706	1075.1**	5003.7	894.7**
G x E	2129	0.38		4		25		5.6	
IPCA 1	156	0.94	3.7**	23	12.1**	128	10.8**	17.3	5.3**
IPCA 2	154	0.68	2.7**	6	3.0**	47	3.9**	10.5	3.2**
IPCA 3	152	0.62	2.4**	5	2.9**	28	2.4**	8.1	2.5**
IPCA 4	150	0.45	1.8**	4	2.2**	23	1.9**	7.6	2.3**
Residuals	1517	0.26		2		12		3.2

### QTL analysis at single environments resulted in low associations

GWA analyses of the four phenotypic traits at the single-trial level produced relatively weak associations. For grain yield, only two MTAs were detected above a Bonferroni threshold of *P*-value < 0.05 (− log_10_(0.05) = 5.75), while 20 MTAs were detected for heading date and none both for plant height and for thousand-grain weight. Both MTAs of GY were detected from ITA10; the 20 MTAs for HD were from ESP16. Lowering the threshold to a more liberal *P*-value < 0.0001, commonly used in GWA studies, still detected 125 MTAs for grain yield, 96 MTAs for heading date, 54 MTAs for plant height, and 10 MTAs for TGW (Table S3). Some regions with common QTLs across trials were suggested, but, overall, the number and strength of associations found was low.

### Meta-analysis identified QTLs with stable effects across environments

**Fig. 2:**
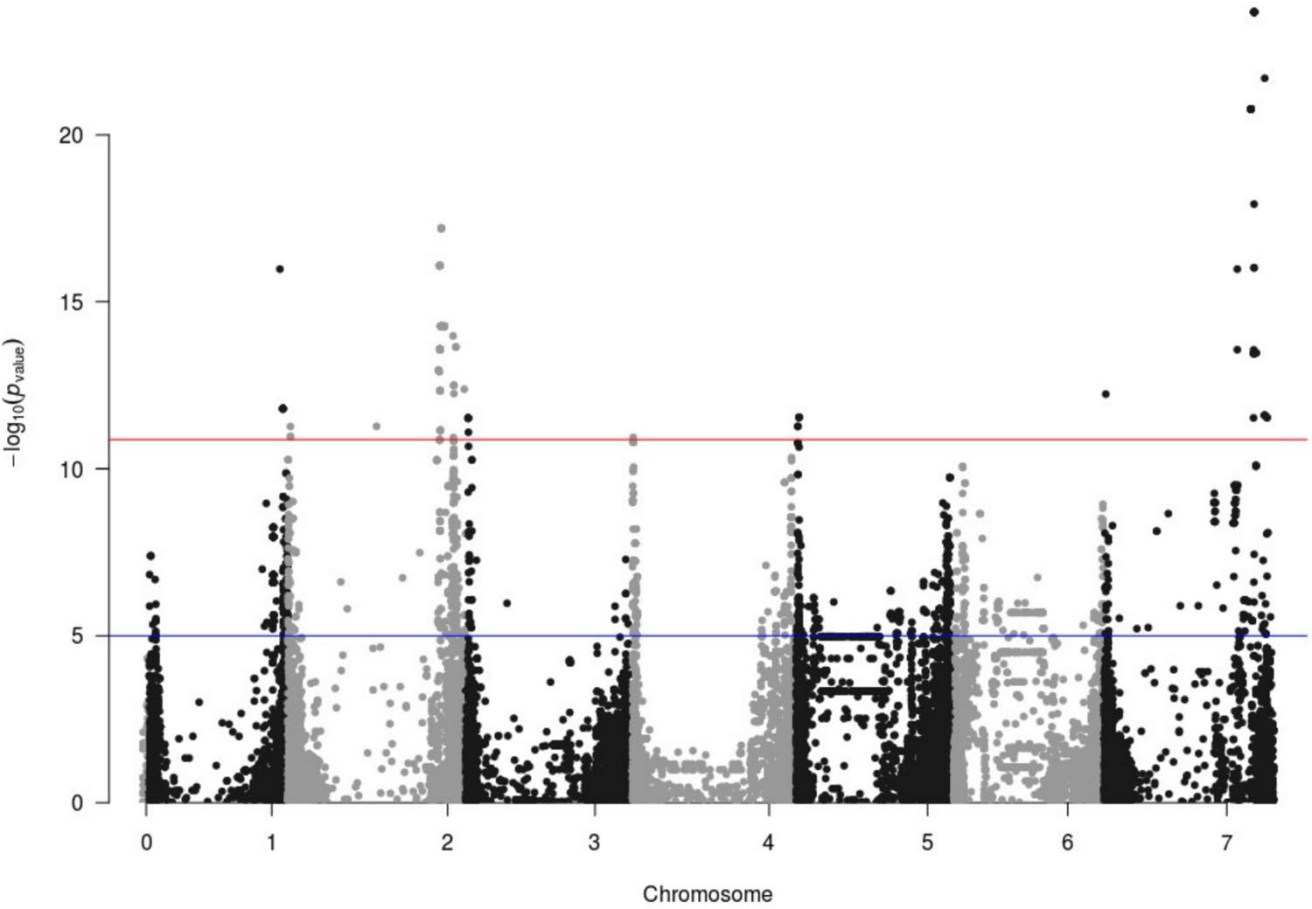
Manhattan plot of the meta-analysis of 14 trials for grain yield (GY). In blue, threshold commonly used in meta-analyses in the literature. In red, threshold calculated in this study, corresponding to the minimum *P*-value resulting from 1000 permutations

The meta-analyses amplified the association signals, based on the relevance of P-values and the commonality in the direction of the effects sign across trials. The results were analysed using a stringent threshold based on permutations. Many SNPs had associations above that threshold. Some markers clearly indicated the same chromosome region. Associated markers were merged into QTLs, combining several criteria. All associated markers in a chromosome were subjected to cluster analysis, as in Looseley et al. (2020), suggesting groups likely belonging to the same QTL. The local LD decay (and chromosomal LD decay, if local LD was indeterminate) helped to delimit the QTLs on each chromosome. This process resulted in the detection of 23 QTLs for heading date, 29 for plant height, 11 for grain yield, and 27 for thousand-grain weight (Table 3, Fig. 2, Fig. S8). These numbers are rather high, because of the detection of QTLs having minor effects (Table S4), which are usually not found in studies of smaller scale.

**Table 3:** Codes, positions, and significance of QTLs detected in the meta-analyses for the four traits studied. The last two columns correspond to the meta-analyses carried out splitting the trials in two subsets, according to the main genotype-by-environment trends found for HD and PH. Flag marker ID starting with “JHI” are trimmed to shorten its length, i.e. JHI-9615 for JHI-Hv50k-2016–9615

Trait	QTL	Flag marker	CHR	Position (bp)	MAF (%)	-log_10_(*P*)	CI Left	CI Right	Frequency shift		
										AUTUMN-log_10_(*P*)	SPRING-log_10_(*P*)
HD	HD1	JHI-9615	1H	9,334,749	7.95	21.59	8,657,247	10,012,251	− 0.0198	4.65	18.09
HD	HD2	JHI-22609	1H	288,026,140	23.18	15.78	227,188,624	348,863,656	0.0278	5.28	11.20
HD	HD3	JHI-30295	1H	393,403,435	8.61	20.22	370,210,929	416,595,941	− 0.0754	12.66	9.74
HD	HD4	JHI-40964	1H	473,573,354	14.57	22.66	463,423,350	483,723,358	0.0833	8.67	14.84
HD	HD5	JHI-79301	2H	48,535,423	21.85	18.87	43,707,921	53,362,925	0.3175	1.69	20.10
HD	HD6	JHI-84334	2H	80,227,961	8.61	13.12	54,440,457	106,015,465	0.1667	1.71	20.41
HD	HD7	JHI-139077	2H	650,766,414	6.62	32.33	648,843,896	652,688,932	− 0.0556	11.91	21.32
HD	HD8	JHI-160692	3H	21,859,520	28.48	17.77	20,147,016	23,572,024	0.3334	1.06	20.39
HD	HD9	JHI-195020	3H	520,711,672	7.28	16.69	516,881,670	524,541,674	− 0.1111	6.36	11.08
HD	HD10	JHI-202588	3H	553,851,218	25.83	18.88	551,156,212	556,546,224	0.0437	6.50	13.12
HD	HD11	JHI-230339	4H	11,284,074	7.95	15.4	9,756,570	12,811,578	− 0.0556	5.14	10.95
HD	HD12	JHI-258528	4H	553,142,475	21.85	16.45	550,707,467	555,577,483	− 0.0873	3.52	13.95
HD	HD13	JHI-274290	4H	605,288,793	8.61	19.89	602,946,289	607,631,297	− 0.0556	6.45	14.19
HD	HD14	JHI-281887	5H	10,837,439	17.22	16.11	9,264,937	12,409,941	− 0.2977	5.62	11.19
HD	HD15	JHI-333135	5H	523,882,465	6.62	21.22	521,534,963	526,229,967	− 0.0873	12.53	10.66
HD	HD16	JHI-363055	5H	580,109,624	5.96	17.67	578,892,122	581,327,125	− 0.0397	5.52	12.89
HD	HD17	JHI-385161	6H	40,578,511	6.62	34.76	9,871,009	71,286,013	− 0.1667	9.33	26.44
HD	HD18	JHI-449563	7H	15,111,871	34.44	28.74	12,271,867	17,951,874	0.1389	8.70	20.89
HD	HD19	JHI-465895	7H	61,645,217	29.14	19.9	55,847,715	67,442,719	− 0.0952	9.40	11.63
HD	HD20	JHI-482284	7H	339,274,267	5.30	20.47	180,054,249	498,494,285	− 0.1111	13.26	9.60
HD	HD21	JHI-487408	7H	445,376,601	5.30	18.13	411,521,597	479,231,605	− 0.0556	13.14	7.80
HD	HD22	JHI-500129	7H	590,186,971	8.61	16.81	586,726,967	593,646,975	− 0.1111	4.80	12.77
HD	HDA1	JHI-36690	1H	439,402,226		9.45	431,402,226	447,402,226		19.67	0.73
										NORTH-log_10_(*P*)	SOUTH-log_10_(*P*)
PH	PH1	JHI-10283	1H	10,222,515	26.49	14.32	8,937,513	11,507,517	0.2659	5.81	7.64
PH	PH2	JHI-26359	1H	341,796,547	21.85	22.67	307,514,031	376,079,063	0.3571	13.09	7.88
PH	PH3	JHI-31326	1H	399,027,554	39.74	22.42	377,665,052	420,390,056	0.6587	12.34	8.20
PH	PH4	JHI-50444	1H	497,490,361	5.30	15.61	496,007,859	498,972,863	0.1667	7.78	7.56
PH	PH5	JHI-71619	2H	21,253,094	28.48	27.93	19,518,088	22,988,100	0.3373	14.13	10.64
PH	PH6	SCRI_RS_207244	2H	43,626,804	25.83	21.73	39,626,802	47,626,806	0.0476	11.06	8.56
PH	PH7	JHI-102401	2H	545,047,467	21.85	11.96	531,869,961	558,224,973	0.2976	4.58	6.83
PH	PH8	JHI-127870	2H	630,365,638	48.34	27.41	627,615,632	633,115,644	0.7857	16.02	10.22
PH	PH9	JHI-149745	3H	2,203,527	5.96	14.65	0	5,218,531	0	7.90	7.67
PH	PH10	JHI-164742	3H	52,796,359	13.25	24.59	24,821,353	80,771,365	0.0834	13.56	10.08
PH	PH11	SCRI_RS_8664	3H	385,814,518	10.60	19.29	232,594,474	539,034,562	− 0.0238	12.43	7.38
PH	PH12	JHI-192298	3H	501,844,817	5.96	14.24	492,514,815	511,174,819	0.0754	9.14	4.40
PH	PH13	JHI-197260	3H	529,944,715	38.41	42.18	521,877,213	538,012,217	0.4524	20.59	17.41
PH	PH14	JHI-204986	3H	562,585,430	29.80	32.94	560,745,422	564,425,438	0.5952	13.25	16.58
PH	PH15	JHI-224935	3H	617,619,009	39.07	17.53	615,594,005	619,644,013	0.504	4.44	8.91
PH	PH16	JHI-233087	4H	28,157,377	17.22	20.19	23,844,871	32,469,883	0.3889	10.20	7.95
PH	PH17	JHI-259677	4H	561,914,675	27.15	13.69	560,192,173	563,637,177	0.1032	5.62	6.22
PH	PH18	SCRI_RS_25685	4H	579,246,000	10.60	25.94	575,945,996	582,546,004	0.1865	15.18	9.10
PH	PH19	JHI-281008	5H	9,003,891	5.96	17.44	8,228,889	9,778,893	0.0556	9.71	7.81
PH	PH20	JHI-325973	5H	510,459,720	23.18	22.05	505,807,218	515,112,222	0.4643	14.83	8.29
PH	PH21	JHI-344172	5H	545,071,584	49.67	14.41	542,706,564	547,436,604	0.6548	6.04	7.00
PH	PH22	JHI-363188	5H	580,264,475	7.95	17.86	579,106,973	581,421,977	− 0.0873	6.24	9.81
PH	PH23	JHI-376336	6H	16,037,775	11.26	20.81	15,397,771	16,677,779	0.0437	12.08	7.24
PH	PH24	JHI-432554	6H	558,950,326	37.09	28.17	553,850,324	564,050,327	− 0.0595	13.31	13.36
PH	PH25	JHI-456995	7H	31,631,859	29.80	23.12	26,229,343	37,034,375	0.246	11.63	8.72
PH	PH26	SCRI_RS_235584	7H	435,303,585	36.42	17.59	404,683,581	465,923,589	− 0.0675	7.33	9.58
PH	PH27	JHI-488571	7H	484,010,786	5.30	12.51	470,178,282	497,843,290	0.0556	6.92	4.42
PH	PH28	JHI-493193	7H	567,606,818	39.07	22.06	562,011,816	573,201,820	0.2778	14.52	5.61
PH	PH29	JHI-501203	7H	593,432,421	5.30	25.17	590,122,417	596,742,424	0.2223	12.88	10.89
TGW	TGW1	JHI-8460	1H	8,528,373	6.62	13.39	7,915,871	9,140,874	-0.0714		
TGW	TGW2	JHI-30897	1H	395,769,146	9.93	10.34	373,764,140	417,774,151	− 0.1508		
TGW	TGW3	JHI-37341	1H	445,816,857	32.45	14.04	433,116,855	458,516,859	− 0.0873		
TGW	TGW4	JHI-78063	2H	43,791,847	6.62	13.59	39,781,845	47,801,846	− 0.1587		
TGW	TGW5	JHI-143015	2H	657,064,597	7.95	22.00	653,724,591.5	660,404,602	− 0.131		
TGW	TGW6	JHI-148635	3H	317,035	13.25	13.01	0	1,259,307	− 0.1865		
TGW	TGW7	JHI-164082	3H	36,220,523	15.89	11.36	31,745,517	40,695,529	− 0.2302		
TGW	TGW8	JHI-186657	3H	467,575,649	45.03	11.72	452,938,141	482,213,156	0.0198		
TGW	TGW9	JHI-199513	3H	539,898,192	6.62	20.74	531,870,684	547,925,700	0.0198		
TGW	TGW10	JHI-224889	3H	616,943,116	5.30	12.63	614,920,612	618,965,619	0.1111		
TGW	TGW11	JHI-230271	4H	11,066,831	17.22	16.95	9,536,827	12,596,834	− 0.0238		
TGW	TGW12	JHI-236625	4H	50,716,395	10.60	14.12	33,296,389	68,136,400	− 0.0953		
TGW	TGW13	SCRI_RS_159331	4H	546,549,711	9.93	13.14	543,149,703	549,949,719	− 0.0873		
TGW	TGW14	JHI-266331	4H	588,890,830	27.15	16.52	585,968,326	591,813,334	− 0.5357		
TGW	TGW15	JHI-281008	5H	9,003,828	5.96	13.76	8,228,826	9,778,830	0.0556		
TGW	TGW16	JHI-308899	5H	441,356,776	27.81	18.68	434,834,272	447,879,279	0.0635		
TGW	TGW17	SCRI_RS_165578	5H	533,639,048	13.91	20.99	531,739,042	535,539,053	− 0.1032		
TGW	TGW18	JHI-364661	5H	582,365,354	41.72	22.95	581,067,852	583,662,855	− 0.4683		
TGW	TGW19	JHI-381643	6H	31,250,013	11.92	13.95	27,115,011	35,385,014	− 0.0119		
TGW	TGW20	JHI-402516	6H	388,187,724	49.67	13.84	359,287,700	417,087,747	− 0.3095		
TGW	TGW21	JHI-433405	6H	560,487,100	17.88	18.04	546,287,100	574,687,100	− 0.0675		
TGW	TGW22	JHI-452562	7H	20,790,898	11.26	13.32	18,458,394	23,123,401	0.1111		
TGW	TGW23	JHI-478583	7H	254,515,002	5.96	13.60	245,715,002	263,315,002	0.0198		
TGW	TGW24	JHI-483539	7H	386,803,631	44.37	10.71	212,081,113	561,526,149	0.1071		
TGW	TGW25	JHI-486280	7H	441,942,697	41.72	18.48	382,762,693	501,122,701	− 0.004		
TGW	TGW26	JHI-488064	7H	479,905,076	41.06	14.31	465,226,728	494,583,424	− 0.2222		
TGW	TGW27	JHI-519440	7H	630,314,109	6.62	15.08	626,879,105	633,749,113	− 0.0159		
GY	GY1	JHI-50447	1H	497,490,053	11.26	11.30	495,987,551	498,992,555	− 0.3571		
GY	GY2	JHI-107800	2H	571,877,011	9.93	15.69	558,557,009	585,197,013	− 0.4286		
GY	GY3	JHI-126025	2H	626,362,758	7.28	12.11	624,117,756	628,607,760	− 0.3214		
GY	GY4	JHI-144055	2H	657,925,477	8.61	11.33	651,895,471	663,955,483	− 0.1667		
GY	GY5	JHI-153479	3H	7,136,438	21.85	11.19	4,503,934	9,768,942	− 0.1389		
GY	GY6	JHI-226878	4H	2,316,928	15.23	9.74	974,426	3,659,430	− 0.4087		
GY	GY7	JHI-279907	5H	6,585,842	31.79	9.86	5,450,840	7,720,844	− 0.9286		
GY	GY8	JHI-441782	7H	6,202,146	12.58	11.76	4,697,144	7,707,146	− 0.2778		
GY	GY9	JHI-489124	7H	496,874,345	5.30	11.39	483,214,341	510,534,349	− 0.0556		
GY	GY10	JHI-492462	7H	559,843,244	11.93	21.14	293,446,961	632,540,561	− 0.0556		
GY	GY11	JHI-504313	7H	598,982,630	8.61	18.35	595,532,628	602,432,632	− 0.2222

**Fig. 3:**
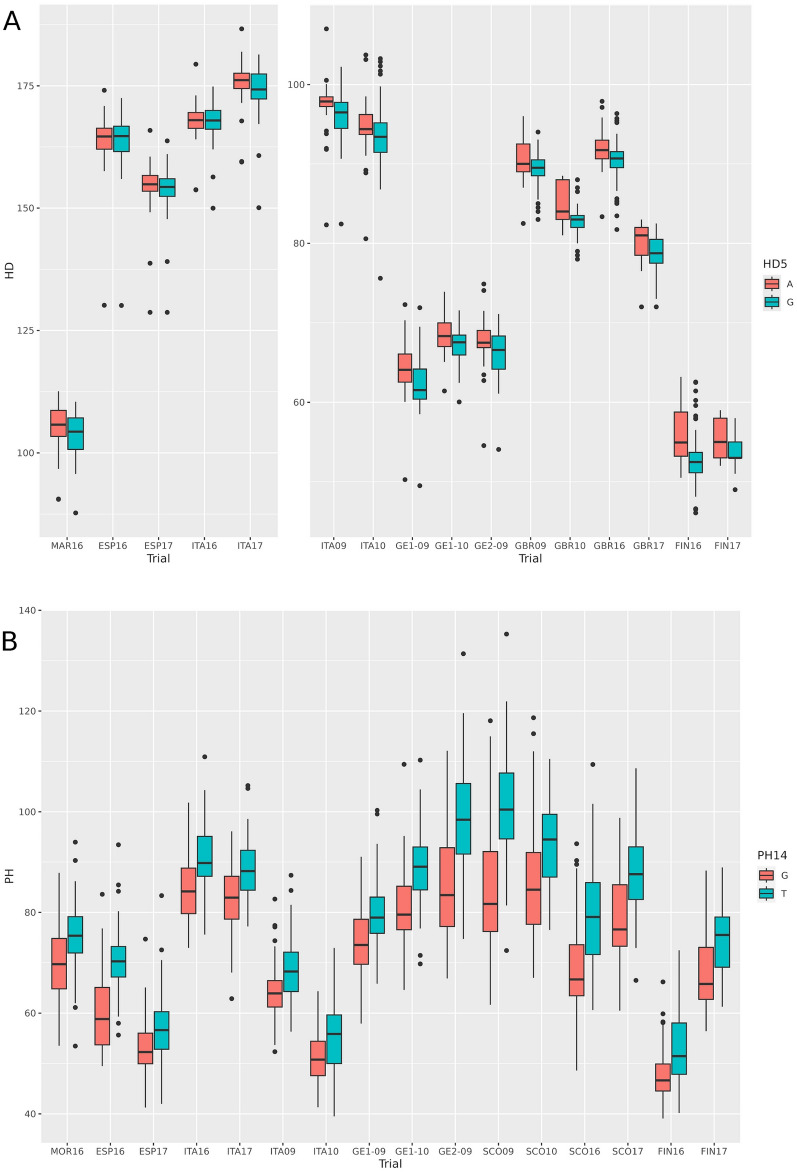

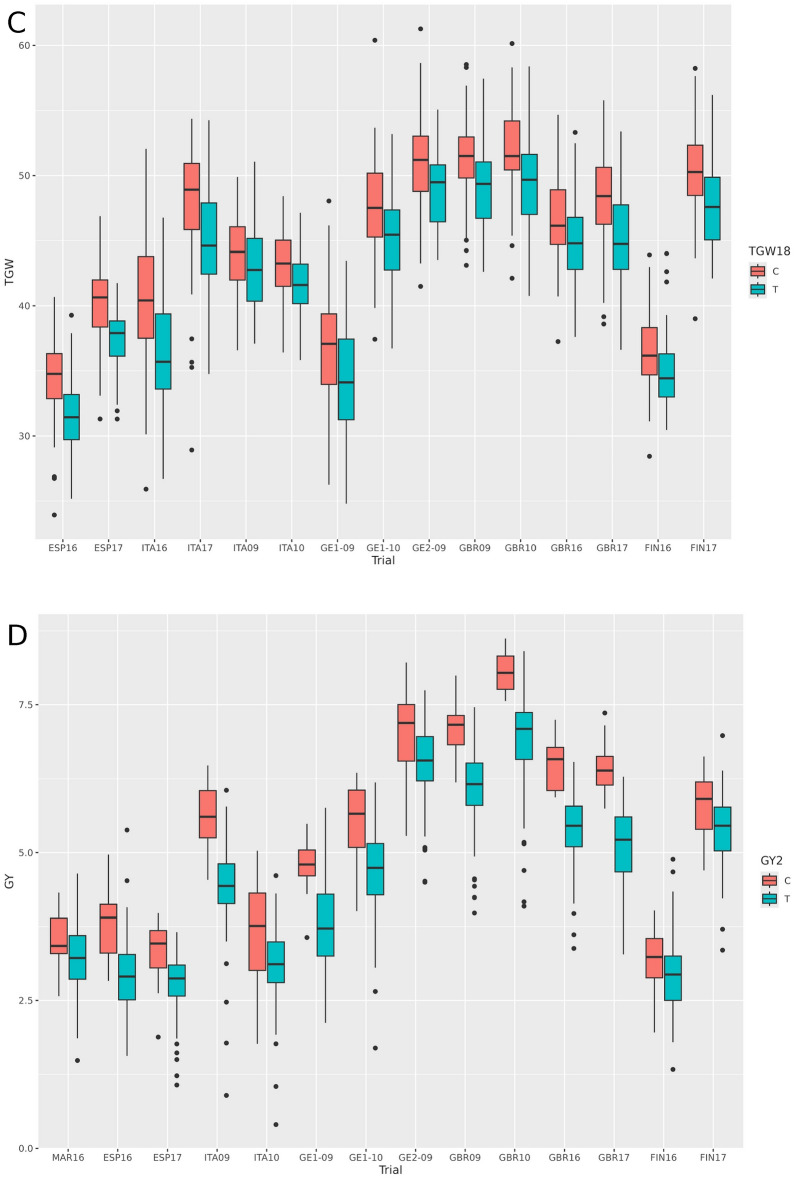
Allelic boxplots for four QTL (one for each trait), across environments. **A** QTL HD5, days from sowing to heading, divided into autumn (left panel) and winter-spring sowings (right panel); **B** QTL PH14, plant height; **C** QTL TGW18, thousand-grain weight; **D** QTL GY2, grain yield

The meta-analysis tends to identify MTAs that show the same sign across all trials. For this reason, it is not expected that it will capture qualitative QTL-by-environment interactions, if these exist. Therefore, most QTLs were rather consistent across trials (Table 3, Table S4). However, some patterns of interaction were evident for a few QTLs, particularly for heading date. The differences in significance between the autumn- and spring-sown trials were large in some cases. The QTLs for HD5 (Fig. 3), HD6, and HD8 were much more significant in spring than in autumn trials. Conversely, HD21 was relatively more important in autumn-sown trials. There was a single QTL detected in the analyses of HD split by sowing time that was not detected in the global analysis. HDA1, on 1H, was significant only in the autumn-sown trials, indicating a marked QTL-by-environment interaction, possibly of qualitative nature, at this locus. The QTL-by-environment interaction was less evident for plant height (Table 3), as shown by PH14 (Fig. 3).

Multilocus models derived from sequential MANOVA analyses were used to identify the best subset of significant QTLs that jointly explained each trait. All QTLs included in the models were significant for *P* < 0.05 or less and had partial Eta squared (η_p_^2^) above 0.14. This value is commonly used as a threshold to declare the influence of independent factors on the dependent variables. These joint models explained around 16% of phenotypic variance for HD, TGW, and GY, and 33% for PH (Table 4). The QTLs retained in the models had a rather small effect, explaining from 1.08 up to 6.07 percent of the variance of the traits: from 0.13 to 0.27 t ha^−1^ (averaged across all environments) for GY, from 0.7 to 1.9 days for HD, 1.4 to 2.7 cm for PH, and 0.6 to 1.3 g for TGW. The QTLs explaining more than 5% of the variance of the trait were PH28, on 7H (2.5 cm), and GY2, on 1H (5.08%, 0.28 t ha^−1^).

**Table 4:** Summary of QTLs kept in the multivariate multilocus analysis for the four studied traits. Best model column indicates the significance of each QTL when included to the model: * as 0.05 ≥ *P*-value > 0.01, ** as 0.01 *P*-value > 0.001, and *** as *P*-value ≤ 0.001

Trait	Chr	QTL	Best model	Partial Eta2	Percentage of variance explained
HD	1H	HD1	*	0.20	1.39
HD	2H	HD5	***	0.37	2.89
HD	3H	HD8	***	0.30	2.23
HD	5H	HD16	***	0.22	1.58
HD	6H	HD17	***	0.26	1.84
HD	7H	HD18	***	0.27	1.95
HD	7H	HD21	***	0.40	3.17
HD	7H	HD22	*	0.21	1.47
					Total HD: 16.52
PH	1H	PH1	***	0.40	3.17
PH	2H	PH6	***	0.30	2.18
PH	2H	PH8	***	0.30	2.23
PH	3H	PH11	**	0.26	1.89
PH	3H	PH13	**	0.23	1.58
PH	3H	PH14	***	0.43	3.45
PH	4H	PH16	**	0.23	1.65
PH	4H	PH17	*	0.21	1.48
PH	5H	PH20	***	0.54	4.72
PH	5H	PH21	**	0.23	1.66
PH	6H	PH23	*	0.20	1.42
PH	7H	PH26	*	0.21	1.50
PH	7H	PH28	***	0.63	6.07
					Total PH: 32.99
TGW	1H	TGW3	*	0.18	1.22
TGW	2H	TGW5	**	0.23	1.63
TGW	4H	TGW14	***	0.39	3.02
TGW	5H	TGW15	***	0.32	2.40
TGW	5H	TGW16	*	0.20	1.36
TGW	5H	TGW18	***	0.26	1.85
TGW	6H	TGW20	*	0.18	1.23
TGW	7H	TGW26	***	0.32	2.41
TGW	7H	TGW27	**	0.21	1.50
					Total TGW: 16.61
GY	2H	GY2	***	0.57	5.08
GY	2H	GY4	**	0.20	1.41
GY	4H	GY6		0.16	1.08
GY	5H	GY7	***	0.30	2.23
GY	7H	GY9	***	0.26	1.86
GY	7H	GY10	*	0.18	1.22
GY	7H	GY11	***	0.39	3.00
					Total GY: 15.88

The intervals for confidence regions of the QTLs were wide, with a mean size of 25.95 Mb. Regions ranged from 1.23 Mb to 349.45 Mb, illustrating just how unevenly distributed is LD in spring barley germplasm. Moreover, two regions of nearly 350 Mb were identified on chromosome 7H, most likely related to an inversion of 141 Mb (Jayakodi et al. 2020) that already has been identified in some cultivars included in our association panel. There was overlap between the confidence intervals for QTLs of different traits, as indicated in Table S5. The region of TGW15-PH19 could represent the same QTL with pleiotropic effects. Some grain yield QTLs overlap with plant height (PH4), and thousand-grain weight (TGW5, TGW25, TGW26, and TGW27). Other QTLs were linked to some extent, which may have implications for their management in breeding.

### Temporal variation in traits and QTL allele frequencies

**Fig. 4:**
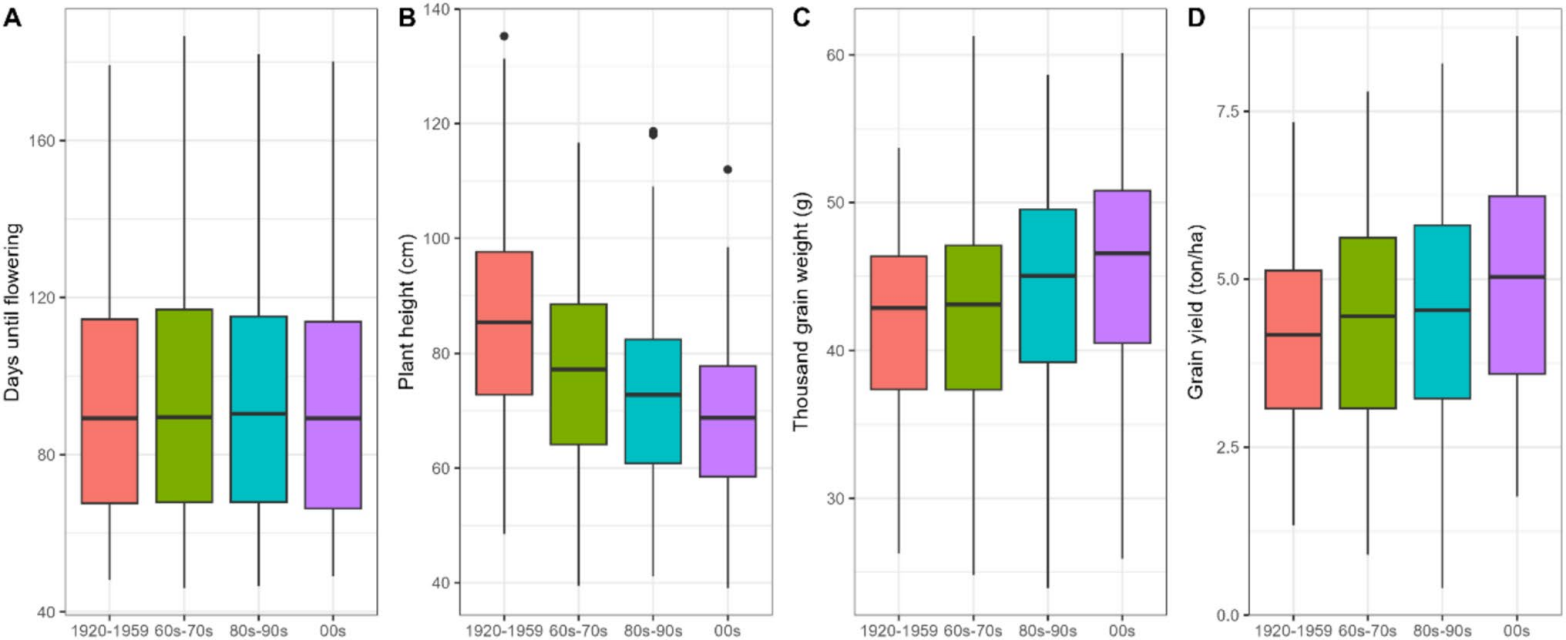
Phenotype boxplots of all trials divided by groups of release years. **A** Days from sowing to heading, **B** plant height, **C** thousand-grain weight and **D** grain yield

The classification of cultivars into four classes, according to the year of release, revealed salient trait and QTL trends resulting from breeding efforts and preferences. Plant height and thousand-grain weight presented marked trends towards reduced (PH) or increased (TGW) values through the years, in accordance with the expected outcome from breeding programs (Fig. 4) and these traits’ heritability (Table 1). The trends were highly consistent across environments (Fig. S4). For grain yield, the increase over time was less marked, except in Scotland. In general, the largest yield improvement was observed for the most modern cultivars compared to earlier ones. There were no marked historic trends regarding heading date. The only remarkable feature was that the most modern class of cultivars was always the earliest in all spring-grown trials, whereas this difference was not observed in the autumn-sown trials, suggesting a possible genotype-by-environment interaction.

**Fig. 5:**
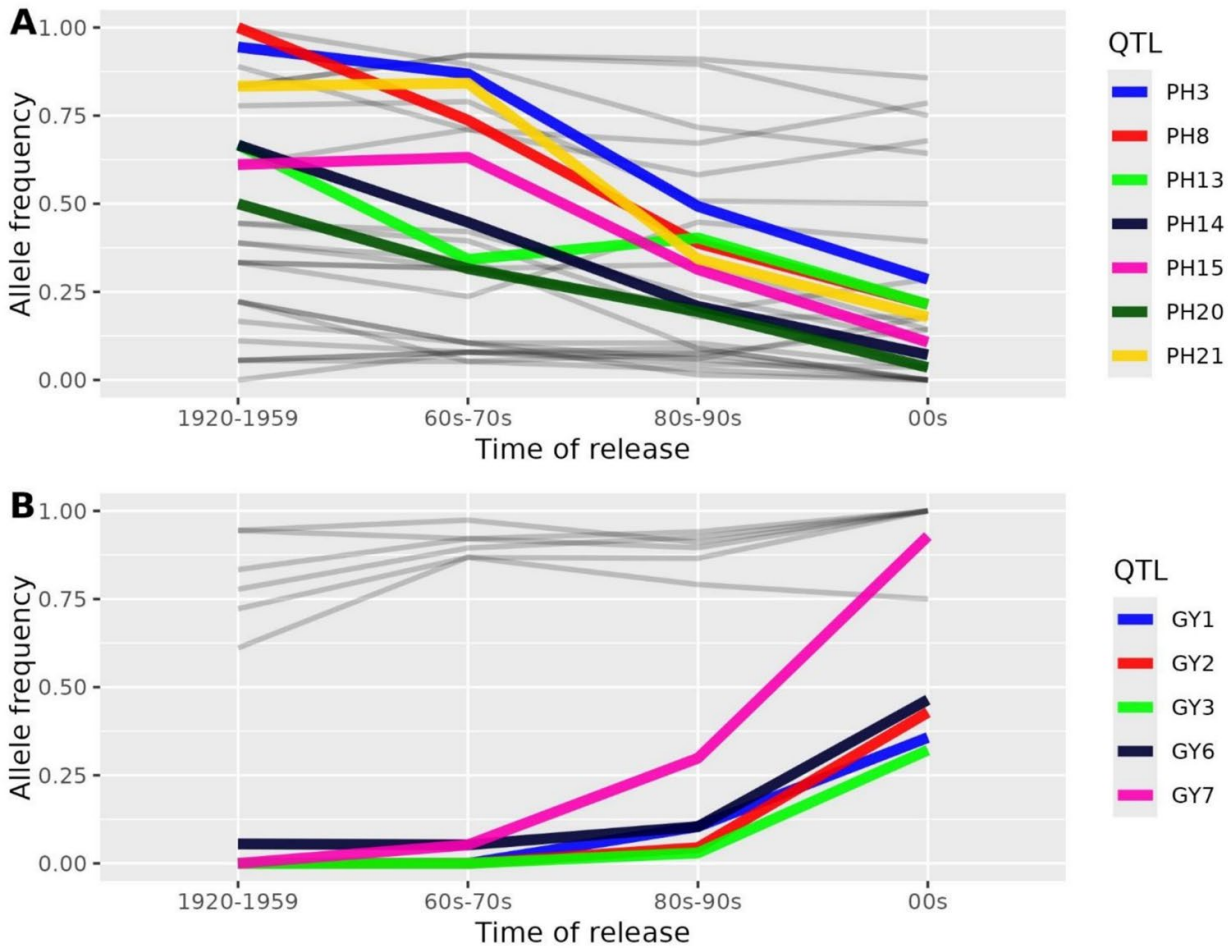
Changes in allele frequencies of the QTLs identified in the meta-analyses for plant height **A** and grain yield **B** across time of release of the cultivars

Regarding allelic frequencies for QTLs over time (Table 3), some QTLs followed the general trend for their trait, while others showed no apparent response. For PH, the height-increasing alleles of eight QTLs were already at low frequencies in the old cultivars; these remained low thereafter. Five QTLs (PH10, PH11, PH17, PH24, PH26) seemed not to be affected by selection, keeping high to very high frequencies (above 0.65) of height-increasing alleles, whereas 11 QTLs exhibited marked increases in the frequency of height-reducing alleles (coloured lines, Fig. 5). For TGW (Fig. S9), the situation was similar, but with fewer QTLs apparently affected by selection. Five QTLs (TGW2, TGW5, TGW6, TGW11, TGW12) showed high frequencies of grain-weight-increasing alleles already in the oldest cultivar class, increasing slightly to almost fixation in the most modern class. The frequencies of another five QTLs (TGW7, TGW14, TGW18, TGW20, TGW26) for higher grain weight approximately doubled over time, i.e. were apparently favoured by selection. For another 17 QTLs, frequencies of favourable alleles varied between medium to low, with little or no response to selection. For GY, the frequency shifts of several QTLs occurred only in the most recent groups of cultivars. An exception was GY7, which was the QTL having the largest frequency change over time, considering all four analysed traits. For HD (Fig. S9), three QTLs were apparently affected by selection (HD5, HD6, and HD8). These QTLs were selected towards earliness. It is remarkable that these three QTLs were detected in the spring-sown trials, yet not in the autumn-sown ones (Table 3), showing the largest difference in significance between the two sets of trials. They may be responsible for the increased earliness shown by the most modern class of cultivars, which is seen only under spring-sown conditions.

At the genome level, some regions seemed preferentially targeted by selection. This was examined by looking at the difference in frequencies between the oldest and newest cultivar classes, calculated for the SNPs divided in 250 rolling windows (bins) per chromosome for all four groups of year of release (Fig. S10). Focusing on the 1% of bins with the largest frequency changes, the most visible selection footprint was in the pericentromeric region of chromosome 5H (65–310 Mb), coincident with the haplotype already detected by Wonneberger et al. (2023). Another narrow region in 5HL (around 543 Mb) showed similar allele frequency changes. Finally, a region on 4HL (510–530 Mb) also showed two close, narrow peaks of very large frequency shifts over time. For all traits, we have identified some QTLs apparently untouched by selection and some at low frequencies in the set studied. These could be aimed at by breeders, provided they have not already been targeted by breeding in recent years and that they do not convey negative pleiotropic effects.

### Exome capture enrichment identified new candidate genes

The inclusion of exome capture (EC) data for the analysis of the QTLs detected provided 119,811 more markers. In 25 cases (31.65%), exome capture markers had equal or higher association than for the 50k SNPs (Table S6); these were designated as the new flag SNPs. For each QTL, a search of candidate genes for the flag markers was carried out within the confidence interval regions. In some cases, flag markers were present inside genes having annotations relevant for the traits considered. The EC markers provided higher resolution than did SNPs from the 50k set, for example, in the region of flowering time QTL HD17 on chromosome 6H. That confidence interval region harbours two flowering-related genes, *HvCMF3* (Cockram et al. 2012) and *HvZTLb* (Russell et al. 2016). However, the EC marker with the largest association is located inside the gene model HORVU.MOREX.r3.6HG0557980, annotated as the nuclear pore complex protein Nup 160.

Several plant height QTLs presented EC markers with higher associations than those of markers in the 50k SNP set. PH1 showed highly associated EC markers in two different genes. While the most strongly associated marker was within a low-confidence gene, the second group of highly associated markers pointed to a nitrate transporter (HORVU.MOREX.r3.1HG0005090), which is an ortholog of rice *OsNRT1.4* (Bucher et al. 2014). PH24, which presents one of the highest associations, was within HORVU.MOREX.r3.6HG0632820, annotated as a WRKY transcription factor-like protein. The *sdw1* gene is a good candidate for QTL PH14. Mutations in this gene, a gibberellin 20-oxidase gene (*HvGA20ox2*), are one of the most common causes of semi-dwarfism in barley. It is a multiallelic locus, with a few alleles commonly employed in barley breeding to reduce plant height (Xu et al. 2017).

Half of the QTLs for thousand-grain weight contained interesting candidate genes, indicated by a higher association of an EC marker. For TGW18, included in the multivariate multilocus model, an ortholog of rice gene *OsBRXL4* was identified (HORVU.MOREX.r3.5HG0535760). The EC flag maker gene for TGW27 is HORVU.MOREX.r3.7HG0752360, which is a lysine-specific demethylase. Interestingly, this is the same annotation of the *Vrs3* gene (Bull et al. 2017; van Esse et al. 2017) and is expressed in developing inflorescences and grains. Its homologous gene in *A. thaliana* is activated under dehydration stress (Huang et al. 2019). For grain yield, enrichment with EC markers identified a gene coding an ɑ-glucosidase enzyme in QTL GY5. This gene, *HvAGL2* (HORVU.MOREX.r3.3HG0221900), was previously confirmed to be involved in starch metabolism in developing grains in barley (Andriotis et al. 2016). The GY2 confidence interval includes the *HvHOX1* (*Vrs1*) gene, involved in spike-row determination (Komatsuda et al. 2007). We used a specifically designed KASP marker (Table S1) to genotype the panel for the *deficiens* allele *Vrs1.t.* None of the cultivars with the unfavourable allele at GY2 carried *Vrs1.t*. Out of the 15 cultivars carrying the favourable allele at QTL GY2, 14 were available to phenotype and genotype. Thirteen of them (all but Forum) carried the *deficiens* allele. An evaluation of the spikes of the 14 revealed differences in the size of the lateral spikelets. Eleven cultivars were phenotypically *deficiens*, without lateral spikelets. Forum was clearly not *deficiens*, genetically and phenotypically. Although Felicitas and Tocada are genetically *deficiens*, both showed very small laterals spikelets (Fig. S11). These results support *Vrs1* as a candidate gene for GY2.

## Discussion

### Meta-analysis improved the identification of associated regions

The sensitivity of the meta-analysis varied among traits. Using the rather liberal threshold (widely found in the literature) of − log_10_(*P*-value) = 4 at the single-trial level, a total of 96 MTAs were detected for HD, in approximately 16 regions, with no QTL detected in more than three trials, while the meta-GWA allowed identification of 22 QTLs, each including several MTAs, in high-confidence regions for HD (reduced to eight in the multilocus analysis). For PH and TGW, only three and six regions, respectively, were detected in single trials (with a maximum coincidence of five trials), much less than those identified by the meta-analysis. For grain yield, there were more regions detected in single trials, altogether 125 MTAs in 20 regions, compared to the 11 QTLs found in the meta-analysis. However, with a threshold of five, the number of MTAs dropped to 48 (in two QTL regions), zero, six (in one QTL), and 34 (in nine QTL regions), for HD, TGW, PH, and GY, respectively**.**

Overall, the meta-analysis increased the power of QTL detection; the confidence of the main-effect QTLs is high, particularly those in the multilocus analyses. The more trials involved, the greater power of detection achieved. We were able to detect QTL effects as low as just above 1% of the traits’ variances. The stringent permutation test which was used protects against false positives. A strict LD control to determine the confidence intervals, and the use of multilocus models, restricted the number of QTLs detected even further, but we still found a high number of QTLs. Besides the increased power of the meta-analysis, breaking down the analysis by the main G x E component detected for HD and PH revealed QTLs and candidate genes specific for certain conditions, such as sowing date (autumn/spring) or latitude. The unique behaviour of cultivars Mona and Saana is likely caused by carrying the same mutant allele in a major flowering time gene, *HvELF3* (Faure et al. 2012; Göransson et al. 2021). This causes them to bypass the photoperiod sensitivity mechanism, leading to early flowering irrespective of the daylength. This fact makes them relatively early in Southern environments, in which most of the growing period occurs under a short photoperiod.

### Associated regions identified with similar association panels

The QTL regions found in this study were compared with those of recent studies involving large populations and SNP genotyping, therefore allowing direct comparison. First, the co-localisation of plant height QTLs with those reported by Tondelli et al. (2013) was rather high. This is not surprising, as our genotype set is a subsample of theirs (they used a 216 spring two-rowed panel), and some trials were also shared (those from 2009 and 2010). We found 29 PH QTLs, compared with 17 found in Tondelli et al. (2013). Out of those 17 PH QTLs, 11 were inside the confidence intervals of our QTLs (PH1, PH2, PH4, PH6, PH11, PH13, PH14, or very close to them PH15, PH20, PH25, PH29). Plant height QTL derived from part of our trials (2016 and 2017) were reported by Bretani et al. (2022). They studied a larger diversity panel, comprising 165 two-rowed (including our entire panel), and 96 six-rowed barleys. They found 48 PH QTL, 26 of them either in the two-rowed or in the whole panel. Twelve of those 26 were inside the confidence intervals of nine of our QTLs (PH2, PH3, PH4, PH5, PH8, PH13, PH14, PH20, PH28). None of our QTLs coincided with their 22 six-rowed specific QTLs.

The same association panel as in Tondelli et al. (2013) was studied by Xu et al. (2018). In this case, only two QTLs, one for grain yield (GY6) and one for thousand-grain weight (TGW18), are shared with our results. This low number of matches could be explained by different methodologies for QTL detection. Multilocation QTL analysis was run using Genstat software, which allows the finding of QTL in interactions with the environment. As mentioned above, the meta-analysis focuses on QTLs with low interaction. Out of the 23 QTLs detected for GY and TGW in the work by Xu et al. (2018), only eight were detected as the main factor QTL (i.e. not interacting with the environment) and one of them coincided with a TGW found in our study.

### MetaQTLs were in common with more complex association panels

Several studies have identified genomic regions associated with the same traits but in completely different association panels or in multi-parent populations. Bustos-Korts et al. (2019) studied a highly diverse panel of 371 genotypes including landraces and cultivars, two- and six-rowed, winter and spring barley. They scored plant height, TGW, and flowering time (Z55) in common with our study. Two QTLs are shared for flowering time (HD12 and HD13), one of them in the region of *HvVRN2*. For plant height, two QTLs have matching confidence intervals (PH8 and PH14); *sdw1* is within one of them. For TGW, there is no QTL in common, although they found a QTL for this trait close to the *Vrs1* gene, which is the same location as our QTL GY2.

Recently, a large association panel (*n* = 363) of European two-rowed spring barley was analysed for flowering time, plant height, and TGW (Bernád et al. 2024). One QTL for flowering time on chromosome 6H had overlapping confidence intervals with HD17. Meanwhile, one MTA for plant height is within PH14, where *sdw1* is located, while another one on chromosome 7H is close to PH25, where their LD block should overlap. For TGW, one of their four QTLs has also been identified in this study (TGW9, chromosome 3H).

We found evidence of surprisingly highly colocalizing QTLs in a recent study addressing two- and six-rowed spring barley germplasm, which was less related to our panel than that of other previous studies. Two articles published using a diverse multi-parent barley population found new QTLs with minor-to-moderate phenotypic effects (Shrestha et al. 2022; Cosenza et al. 2024). Several of our HD QTLs co-located with those found in Cosenza et al. (2024): HD3, HD4, HD10, HD15, and HD17 had overlapping confidence intervals with QTLs found in the multi-parent population analysis, whereas QTLs overlapping with HD5, HD13 and HD22 were detected in single populations. For plant height, 14 of the 29 QTLs (Table 3) co-located with QTLs for the same trait in the multi-parent population. Most of them were detected in single populations, but three (PH15, PH22, PH24) were detected in the whole population. Regarding TGW, out of the 27 QTLs found in our study, 10 coincided with TGW QTLs in the multi-parent population (Shrestha et al. 2022).

The population studied by Shrestha et al. (2022) and Cosenza et al. (2024) is composed of two-row and six-rowed subpopulations and, thus, probably represents a larger genetic diversity space than does our panel. Moreover, the multi-parent population they used is more amenable to the detection of small effect QTLs. In any case, the multiple shared locations for QTLs in our study and theirs are likely a result of the higher power of detection achieved with either approach, thus providing further confidence in the associations found in the present study. Therefore, the loci identified constitute a sound set of QTLs, with a clear potential to contribute to barley breeding in Europe.

A recent study (Hong et al. 2024), using a large and diverse two-rowed barley panel, found 38 MTAs for grain size, one in common with our TGW20 QTL. Finally, a meta-analysis revising yield-related traits of 54 studies that were published since 2000 (Du et al. 2024) found four QTLs co-locating with ours: TGW3, TGW17, TGW25 and GY2.

### Implications for breeding

The genome-wide allele frequency changes have identified major selection footprints caused by barley breeders during the last century. We concentrated only on the most salient selective sweeps, although it is evident that breeding affected allele frequencies throughout the genome, the shifts (large or small) paralleling always the time gradient (Fig. S10). Those genomic regions included QTLs related to disease resistance. The pericentromeric region of 5H, and its possible relation to the introduction of leaf rust tolerance, was thoroughly described in Wonneberger et al. (2023). Interestingly, the narrower, but equally strong, selection footprint of 5HL appears to be associated with two stem rust resistance QTLs (Case et al. 2018). The selection footprint found in 4HL was not associated with agronomic QTLs. The closest one was TGW13, 15 Mb away. However, a QTL for net blotch tolerance, on the position of the selection footprints, was found by Daba et al. (2019).

The dynamics of the evolution of allele frequencies for the agronomic QTLs in the timeframe of our study suggest an early selection of many plant height QTLs, followed closely in time by selection for thousand-grain weight. Not surprisingly, both traits usually show high heritability. Effective selection for grain yield QTLs occurred later, apparently facilitated once the variance for the other traits was reduced. Plant height is easy to select, due to its high heritability, and shortness was preferred to minimize lodging, particularly after the introduction of semi-dwarf alleles like *denso*/*sdw1* (detected as PH14). Grain yield and thousand-grain weight were also important breeding targets for production and quality, but their selection appeared stronger after PH was improved. For instance, in the close QTL pairs PH1-TGW1, PH6-TGW4, PH15-TGW10, PH4-GY1, and PH8-GY3 (Table S5), PH-favourable alleles were fixed, or almost so, in the most modern class of cultivars, whereas the frequencies of favourable alleles for the QTLs of the other trait were low (although already growing in some cases). This was also the case for the region with QTL PH27-TGW26-GY9. In this region, in modern varieties favourable alleles for GY and PH are almost fixed, while the favourable TGW allele frequency is still low. In other cases, favourable alleles for the two traits in a particular region were found in intermediate frequencies throughout the breeding history (PH10-TGW7, PH26-TGW25, PH13-TGW9, PH24-TGW21). Therefore, selection for both favourable alleles is likely feasible although, in the last two cases, the genotypic frequencies indicate a possible linkage in repulsion for the favourable alleles. In all other cases, the favourable alleles have already been selected. These observations can be of direct use for barley breeders.

The largest frequency shift across time of all detected QTLs in this study, around 0.9, was identified in GY7. It was very effectively selected throughout the second half of the twentieth century. The QTLs PH19 and TGW15 lie close to GY7. These two QTLs share the flag marker, although with opposite effects for agronomic fitness. One allele is associated with low height and thin grains, and the other one with tall plants and large grains. The antagonistic effect of a single gene on these two traits was already described for the main semi-dwarfing gene in barley, *HvGA20ox2* (Thomas et al. 1991). In this case, breeding selected short plants preferentially over large grains, although grain weight may have been compensated by selection at other loci. A possible explanation of the historic trends observed is that, once the PH19/TGW15 QTL was almost fixed for short plants, selection for grain yield at this region was facilitated. Indeed, favourable alleles for low TGW and plant height appear together in 142 cultivars, whereas the only nine cultivars with the “elevated height” allele also present the “high TGW” allele.

The near fixation of favourable alleles for traits which suffered strong selection pressure on neighbouring QTLs may have helped the selection at GY7. A similar situation may have occurred at 5HL, where the near fixation of the favourable (low height) allele at PH22 may have strengthened selection near TGW18, whose favourable allele suffered strong selection over time. The proximity of some QTLs and their fates throughout breeding suggest possible targets for future breeding. For example, in the distal region of 3HL, PH15 shows a decreasing frequency of “high” alleles, indicating selection pressure; however, the favourable allele of the neighbouring QTL, TGW10, remains at a low frequency. This suggests the presence of linked favourable alleles in repulsion, a situation that could be addressed by breeding. A similar phenomenon occurs at the beginning of 2H, with QTL PH6 and TGW4.

### Candidate genes for agronomic traits

Some candidate loci corresponded to the already-known functions of characterised genes, while loci either provided new information about the effects of known genes or revealed genes with unknown roles in barley, but with suggested ones based on orthologues in rice (Table 5). For example, the candidate gene for HD17 is a homologue of *Arabidopsis thaliana AtNUP160*, associated with flowering time (Li et al. 2020). It anchors HOS1 to the ubiquitinated CONSTANS protein, which has not been reported in barley so far. Further evidence for the possible involvement of this gene in flowering comes from its preferential expression in barley apical meristems, inflorescences, and microspores (Li et al. 2023).

**Table 5:** Best candidate genes found for selected QTLs

Trait	QTL	CHR	Candidate gene	Gene model ID	MorexV3 Annotation
HD	HD1	1H	WNK3	HORVU.MOREX.r3.1HG0004150	Kinase family protein
HD	HD2	1H	HvCMF10	HORVU.MOREX.r3.1HG0041150	Zinc finger protein CONSTANS
HD	HD3	1H	HvCO9	HORVU.MOREX.r3.1HG0058180	CONSTANS-like protein
HD	HD5	2H	HvCO18	HORVU.MOREX.r3.2HG0115000	CONSTANS-like protein
HD	HD9	3H	HvFDL-H4	HORVU.MOREX.r3.3HG0296180	BZIP transcription factor
HD	HD16	5H	HvFRI	HORVU.MOREX.r3.5HG0534990	FRIGIDA-like protein, putative
HD	HD17	6H	HvNUP160	HORVU.MOREX.r3.6HG0557980	Nuclear pore complex protein Nup 160
HD	HD21	7H	HvMADS26	HORVU.MOREX.r3.7HG0705340	MADS-box transcription factor
HD	HD22	7H	HvNF-Yb2	HORVU.MOREX.r3.7HG0734470	Nuclear transcription factor Y subunit B
PH	PH1	1H	HvNRT	HORVU.MOREX.r3.1HG0005090	Nitrate transporter 1.1
PH	PH3	1H	OsCKX9	HORVU.MOREX.r3.1HG0059670	Cytokinin oxidase/dehydrogenase
PH	PH4	1H	HvGA2ox8a	HORVU.MOREX.r3.1HG0087020	Gibberellin 2-oxidase
PH	PH13	3H	OsWRKY21	HORVU.MOREX.r3.3HG0297540	WRKY transcription factor
				HORVU.MOREX.r3.3HG0297550	WRKY transcription factor
PH	PH14	3H	HvGA20ox2 (sdw1/denso)	HORVU.MOREX.r3.3HG0307130	Gibberellin 20 oxidase
PH	PH20	5H	OsPH9	HORVU.MOREX.r3.5HG0496220	Histone H4
PH	PH24	6H	HvLAZY1	HORVU.MOREX.r3.6HG0632820	WRKY transcription factor-like protein
PH	PH29	7H	OsMYB45	HORVU.MOREX.r3.7HG0736780	Homeodomain-like superfamily protein
				HORVU.MOREX.r3.7HG0736790	Homeodomain-like superfamily protein
				HORVU.MOREX.r3.7HG0736800	Two-component response regulator
				HORVU.MOREX.r3.7HG0736840	MYB transcription factor-like
TGW	TGW2	1H	OsCKX9	HORVU.MOREX.r3.1HG0059670	Cytokinin oxidase/dehydrogenase
TGW	TGW2	1H	HvSMOS1	HORVU.MOREX.r3.1HG0058550	AP2-like ethylene-responsive transcription factor
TGW	TGW7	3H	HvRA2 (vrs4)	HORVU.MOREX.r3.3HG0233930	LOB domain protein
TGW	TGW8	3H	HvBRI1	HORVU.MOREX.r3.3HG0285210	Receptor kinase
TGW	TGW13	4H	HvGT1	HORVU.MOREX.r3.4HG0399240	Homeobox protein, putative
TGW	TGW14	4H	HvCMF4	HORVU.MOREX.r3.4HG0411680	Zinc finger protein CONSTANS
TGW	TGW18	5H	HvBRXL4	HORVU.MOREX.r3.5HG0535760	Protein BREVIS RADIX
TGW	TGW27	7H	HvJMJ	HORVU.MOREX.r3.7HG0752360	Lysine-specific demethylase
GY	GY1	1H	HvGA2ox8a	HORVU.MOREX.r3.1HG0087020	Gibberellin 2-oxidase
GY	GY2	2H	HvHOX1	HORVU.MOREX.r3.2HG0184740	Homeobox leucine zipper protein
GY	GY5	3H	HvAGL2	HORVU.MOREX.r3.3HG0221900	Alpha-glucosidase
GY	GY8	7H	HvKAO1	HORVU.MOREX.r3.7HG0637750	Cytochrome P450
GY	GY11	7H	OsMYB45	HORVU.MOREX.r3.7HG0736780	Homeodomain-like superfamily protein
				HORVU.MOREX.r3.7HG0736790	Homeodomain-like superfamily protein
				HORVU.MOREX.r3.7HG0736800	Two-component response regulator
				HORVU.MOREX.r3.7HG0736840	MYB transcription factor-like

Looking at the confidence intervals of HD QTLs, several known flowering-related genes were located within them. Among these 22 QTLs, the confidence regions included *HvCO9* (Cockram et al. 2012), *HvFT3* (Faure et al. 2007; Kikuchi et al. 2009), *HvHAP3* (Campoli et al. 2013), and *HvVRN2* (Karsai et al. 2005), respectively in HD3, HD4, HD6, and HD13. However, *HvFT3* is not a good candidate, since all 151 cultivars share the same (presence) allele in this gene. Similarly, *HvVRN2* is not a likely candidate, as the allelic segregation for this gene does not coincide with that of the QTL (Table S1). On the contrary, three more QTLs were located near flowering-related genes that are good candidates: HD13 was only 3 Mb apart from *HvFT5* (Faure et al. 2007; Kikuchi et al. 2009), HD21 was 5.5 Mb away from *HvMADS26* (Pankin et al. 2018; Hill et al. 2019), and HD16 was 383 kb away of *HvFRI* (Campoli et al. 2013).

The candidate gene for plant height QTL PH24 corresponds to the orthologous one in rice, *LAI1/LAZY1*. This gene regulates the expression of auxin transporters to control tiller angle and shoot gravitropism (Li et al. 2007; Zhu et al. 2020). Interestingly, a candidate gene for the thousand-grain weight QTL TGW18 is the orthologue of rice *OsBRXL4,* which is a regulator of the nuclear localization gene of rice *LAI1/LAZY1* (Li et al. 2019). Although neither rice gene has been studied for grain weight, they are highly related to plant architecture. There is a possible candidate locus affecting two QTLs on 7HL, PH29, and GY11. The candidate is a cluster of four orthologues of the rice gene *OsMPH1*/*OsMYB45*, located exactly in the overlapping interval of the two QTLs. This rice gene affects plant height and grain yield (Zhang et al. 2017), which is consistent with our observations. The favourable alleles at these two QTL were present at high frequencies, and both increased over time until fixation, indicating joint selection in this region.

The *HvHOX1* (*Vrs1)* gene is a candidate for QTL GY2. The haplotype *Vrs1.t*, commonly known as *deficiens* (Sakuma et al. 2017), could underlie the favourable allele, providing a yield advantage of 0.28 t ha^−1^ across all trials. The *Vrs1.t1* allele is a mutant allele of the two-rowed type allele *Vrs1.b2*. Selection at this allele apparently started in the 1980–1990s and was subject to strong selection pressure. This allele conferred larger grains, although this did not result in a yield advantage in the study by Sakuma et al (2017). Those authors hypothesized that *deficiens* induces larger grains by the suppression of organs (lateral florets), not specifically involved in sink/source relationships. In our study, however, this QTL did not show a clear signal for TGW, but the GY response was very consistent across environments. To the best of our knowledge, this is the first time that a yield advantage has been reported for this gene under field conditions.

In summary, our results indicate when and where breeding efforts reduced allelic diversity, which traits were more accessible to breeders, where there were effects on nearby genes, and how. When combined with candidate gene identification, our approach allows the biological function of genes under relevant field conditions to be examined. This information, in sum, provides a road map to assist breeders to more accurately pursue their targets for the future.

## Supplementary Information

Below is the link to the electronic supplementary material.

Supplementary file1 (PDF 2843 KB)

Supplementary file2 (XLSX 319 KB)

Supplementary file3 (XLSX 137 KB)

Supplementary file4 (ZIP 342236 KB)

## Data Availability

The phenotypic and genotypic data that support the findings of this study are provided as supplementary Tables S7 and S8, respectively. Raw exome capture data used in this study is available in the European Nucleotide Archive (ENA) under study accessions PRJEB14445 (Mascher et al. 2017), PRJEB53544 (Chen et al. 2022) and PRJEB88448 (this study). A full list of individual samples’ accession numbers is available in Suppl. Table S1.
