# Supplementary material for: New loci and candidate genes in spring two-rowed barley detected through meta-analysis of a field trial European network
Source: Theor Appl Genet. 2025 Jun 23;138(7):158. doi: 10.1007/s00122-025-04934-8 (PMC12185650; doi:10.1007/s00122-025-04934-8)
Supplement: Supplementary file 1 — Supplementary file1 (PDF 2843 KB) [file 122_2025_4934_MOESM1_ESM.pdf]

New loci and candidate genes in spring two-rowed barley detected through meta-analysis of a field trial European network

Francesc Montardit-Tarda[1], Ana M Casas[1], William TB Thomas[2], Florian Schnaithmann[3], Rajiv Sharma[4,5], Salar Shaaf[4,6], Chiara Campoli[2], Joanne Russell[2], Luke Ramsay[2], Micha M Bayer[2], Stefano Delbono[7], Marko Jääskeläinen[8,9], Maitry Paul[8,9], Frederick L Stoddard[9,10], Andrea Visioni[11], Andrew J Flavell[12], Klaus Pillen[3], Benjamin Kilian[4,13], Andreas Graner[4], Laura Rossini[6], Robbie Waugh[2], Luigi Cattivelli[7], Alan H Schulman[8,9,10], Alessandro Tondelli[7], Ernesto Igartua[1,*]

[1]Estación Experimental de Aula Dei – Consejo Superior de Investigaciones Científicas (EEAD-CSIC), Avenida Montañana 1005, 500059, Zaragoza, España

[2]James Hutton Institute, Errol Road, Invergowrie, Dundee DD2 5DA, United Kingdom

[3]Martin-Luther-Univ. Halle-Wittenberg, Betty-Heimann-Str. 3, 06120 Halle (Saale), Germany

[4]Leibniz Institute of Plant Genetics and Crop Plant Reasearch (IPK), Corrensstrasse 3, 06466 Gatersleben, Germany

[5]Scotland's Rural College, Peter Wilson Building, The King's Buildings, West Mains Road, Edinburgh EH9 3JG, United Kingdom

[6]Università degli Studi di Milano, Via Celoria 2, 20133 Milano, Italy

[7]Council for Agricultural Research and Economics (CREA), Research Centre for Genomics and Bionformatics, Via San Protaso 302, 29017 Fiorenzuola d'Arda, Italy

[8]Institute of Biotechnology, University of Helsinki, FI-00014 Helsinki, Finland

[9]Viikki Plant Sciences Centre, University of Helsinki, FI-00014 Helsinki, Finland

[10]Natural Resources Institute Finland (LUKE), FI-00014 Helsinki, Finland

[11]International Center for Agricultural Research in the Dry Areas (ICARDA), Rabat 10100, Morocco

[12]Dundee University at SCRI, Invergowrie, Dundee DD2 5DA, United Kingdom

[13]Global Crop Diversity Trust, Platz Der Vereinten Nationen 7, 53113 Bonn, Germany

[*]Correspondence: igartua@eead.csic.es

Supplementary Figures

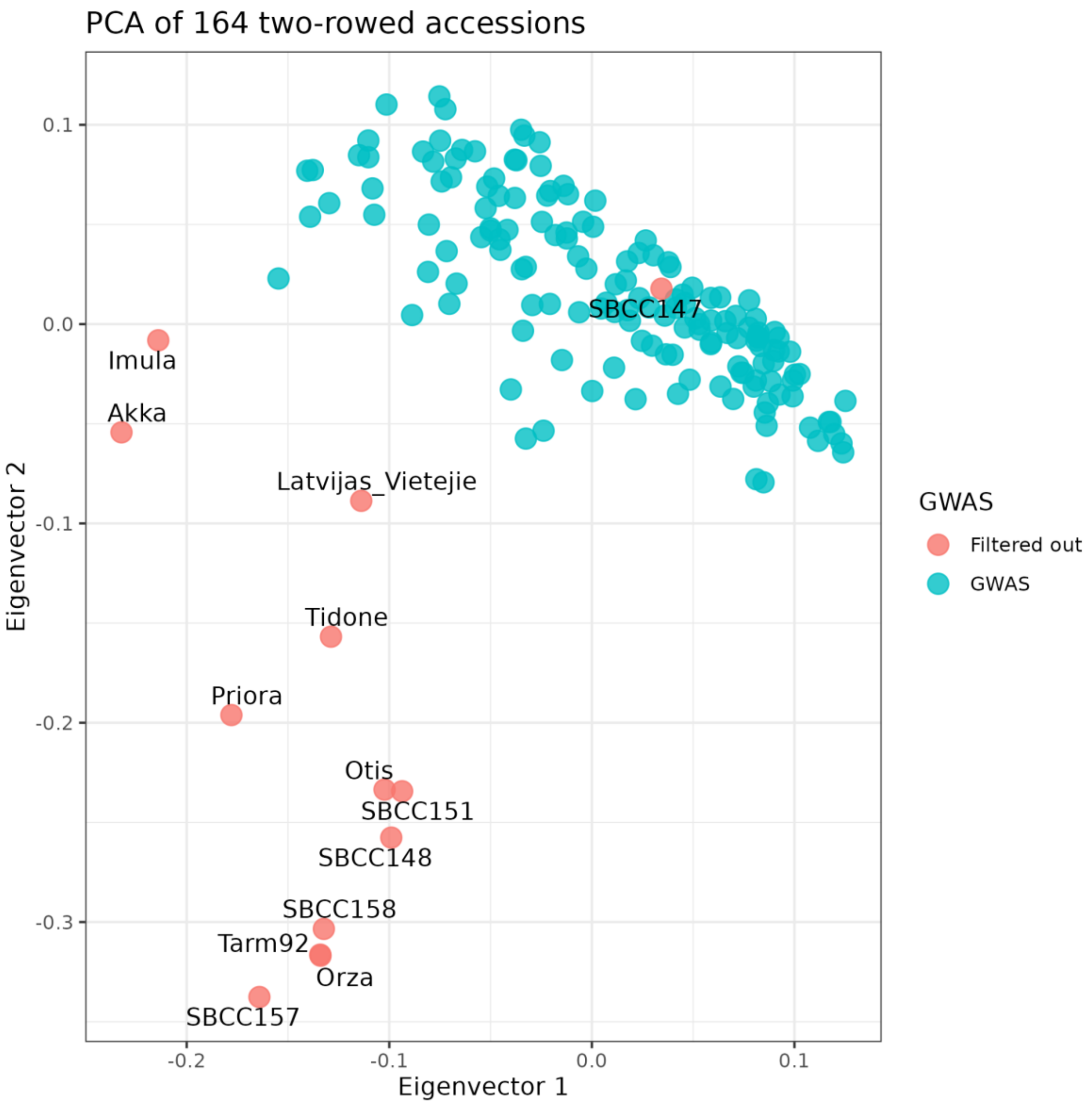

Figure S1. Principal component analysis of spring two-rowed barleys genotyped with the 50k barley SNP chip. Cultivars excluded from the analysis to homogenize population structure are indicated by colour and labels.

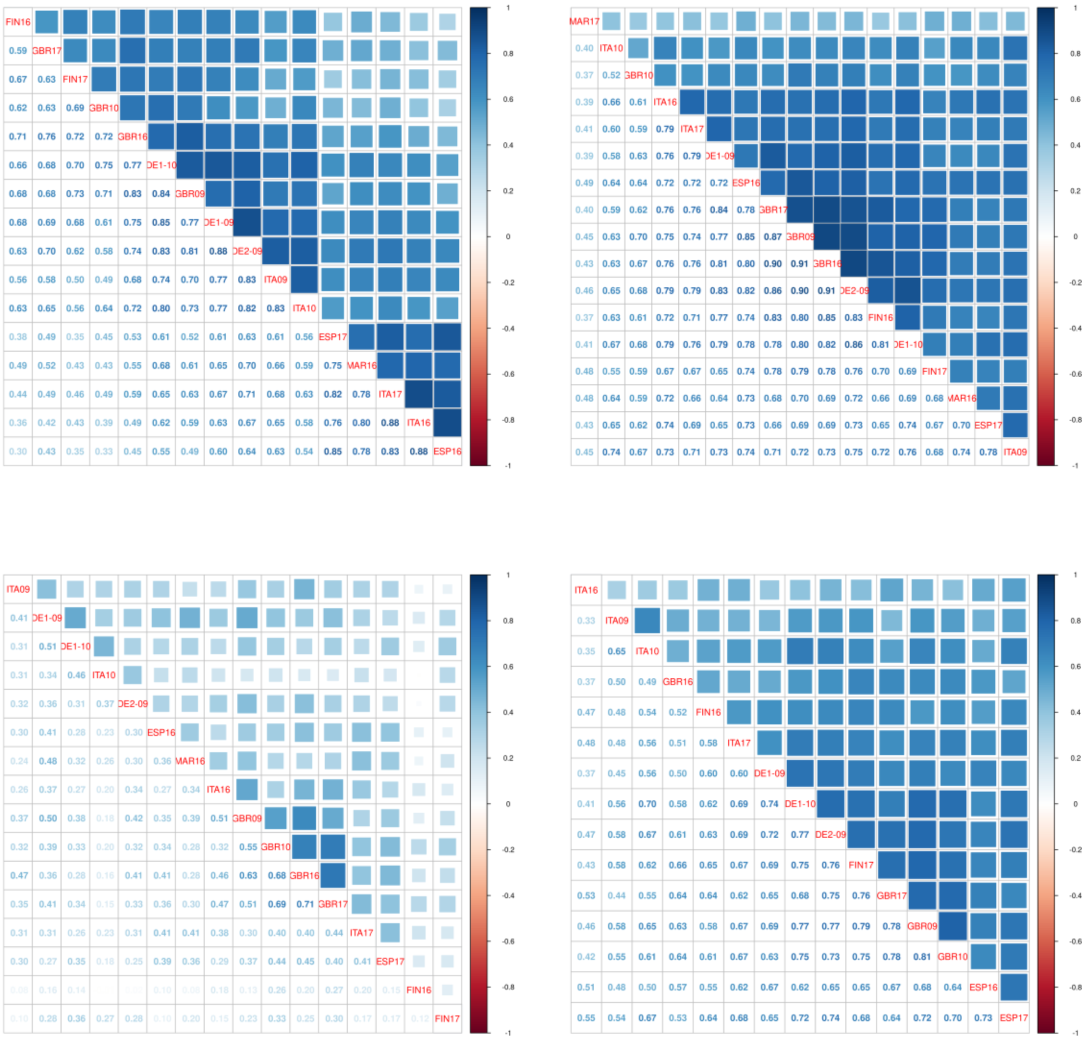

Figure S2. Correlation of genotypic BLUEs between trials. Upper-left: HD, upper-right: PH, bottom-right: GY, bottom-left: TGW. The trial MAR17 was excluded of all GWA analyses because its plant height correlation showed values below 0.5 with all the other trials.

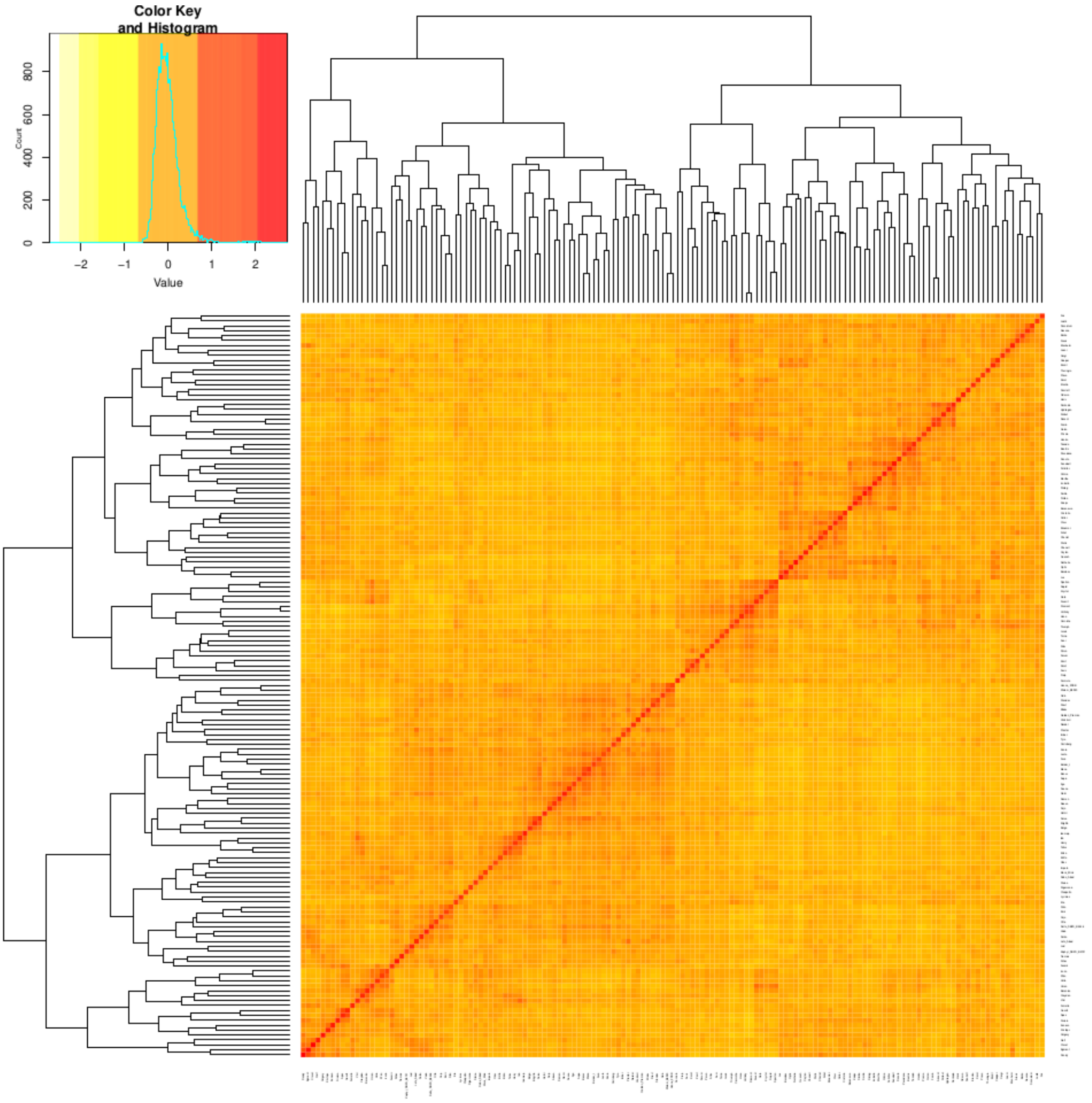

Figure S3. Kinship matrix of the 151 cultivars used as a population structure adjustment in the single-trial GWA analyses.

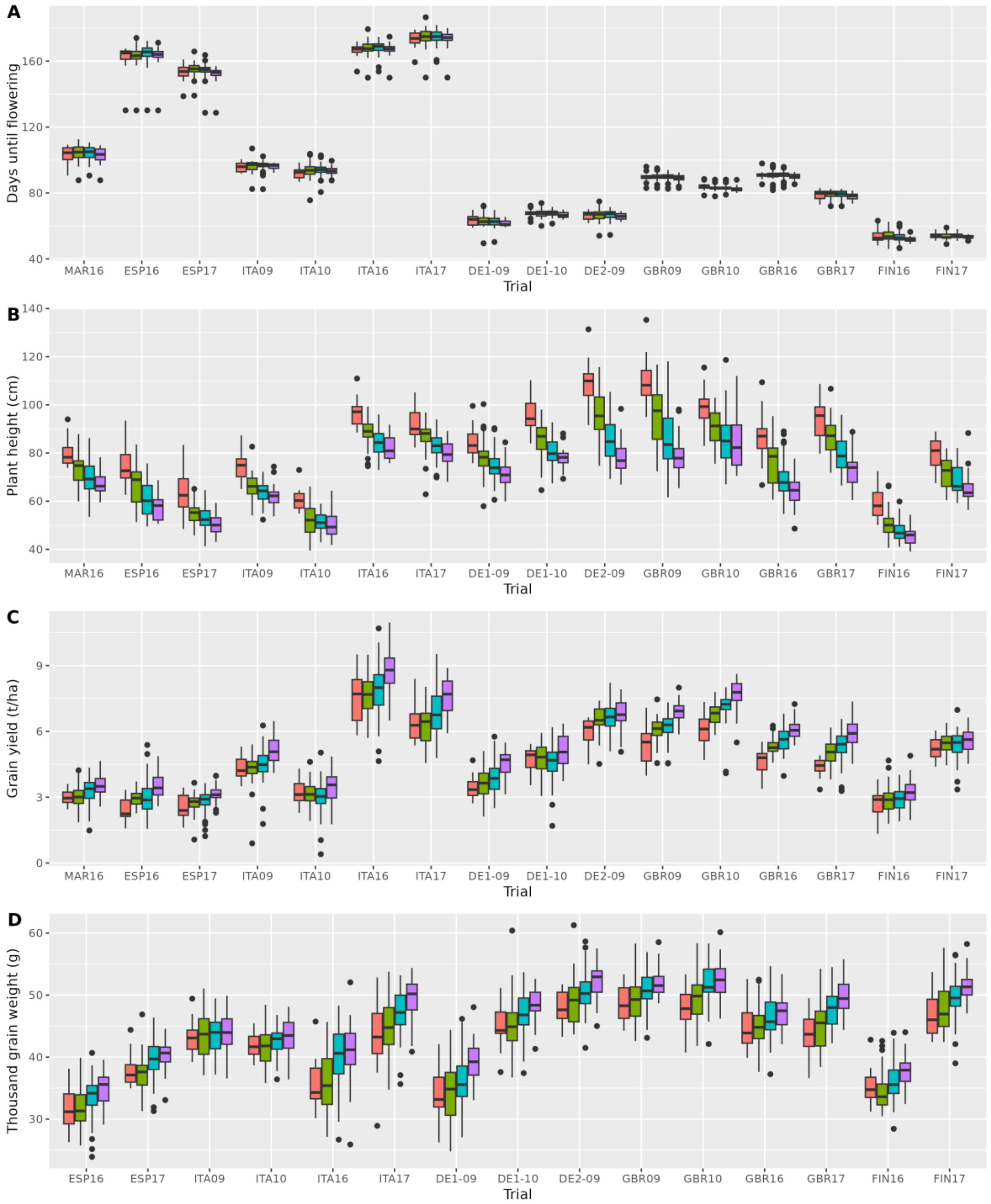

Figure S4. Boxplots of HD (A), PH (B), GY (C) and TGW (D) across trials and year of release.

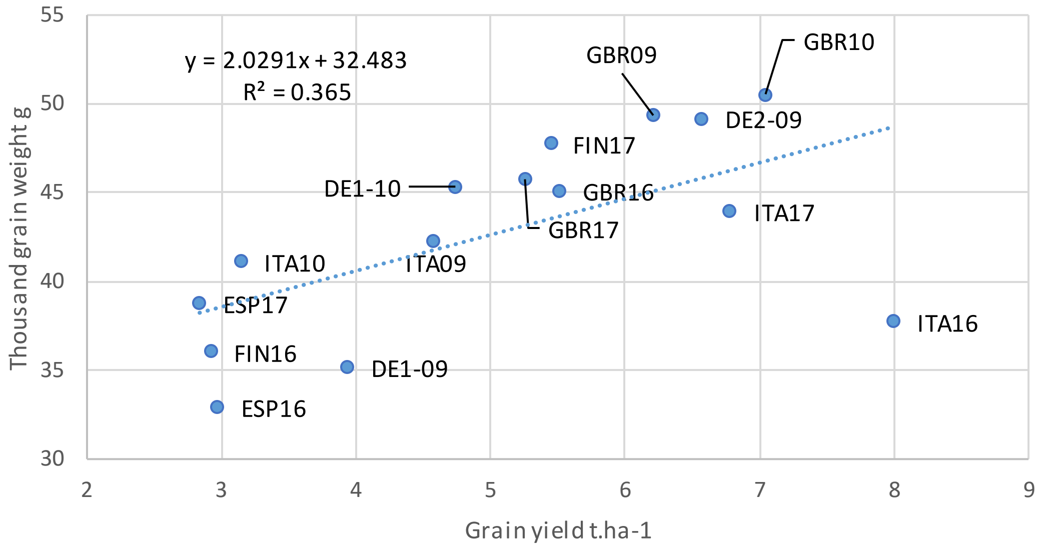

Figure S5. Linear relationships of grain yield with plant height and thousand-grain weight across environments.

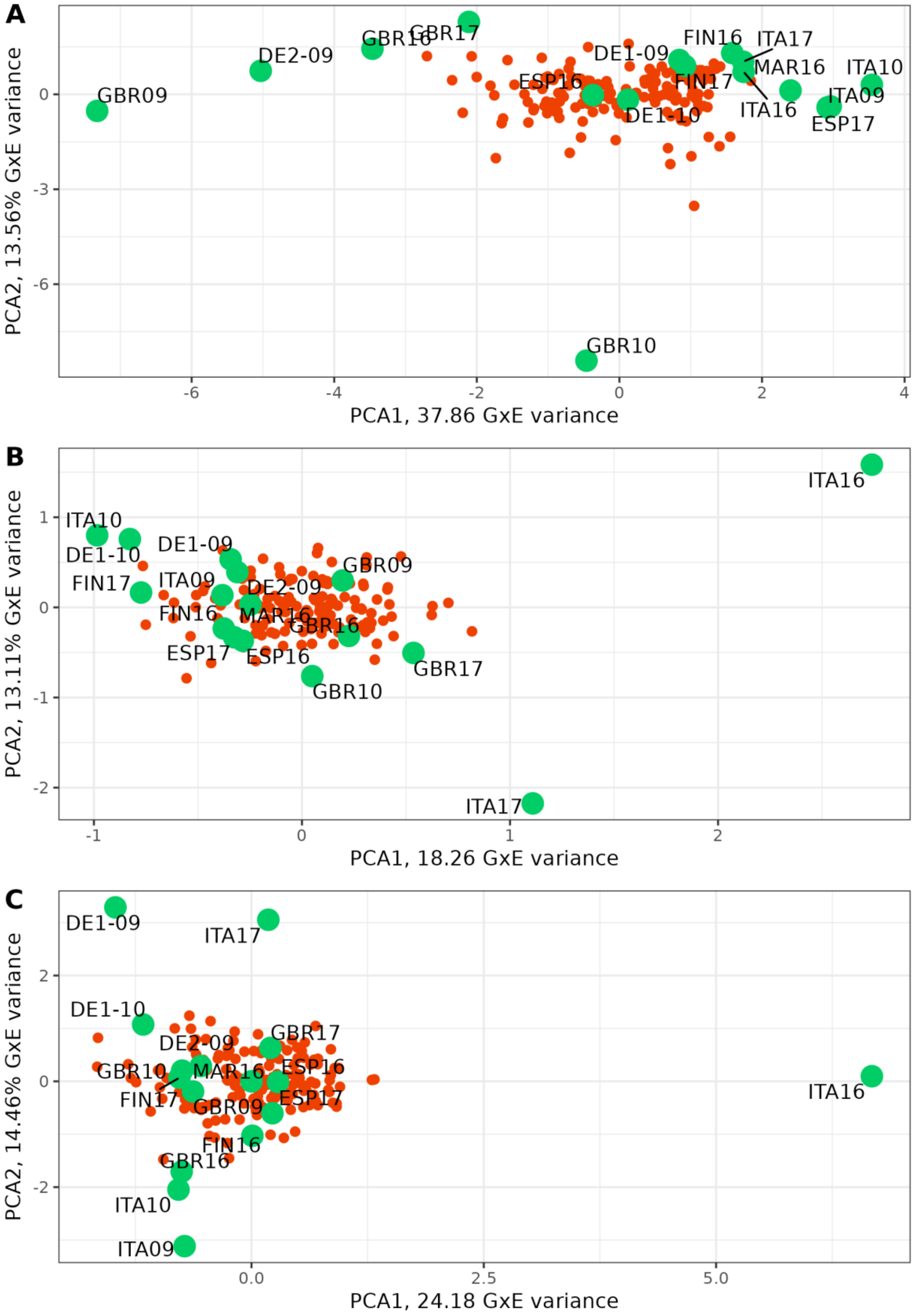

Figure S6. Plots of the first two principal components of the AMMI analyses for A) plant height, B) grain yield, and C) thousand-grain weight.

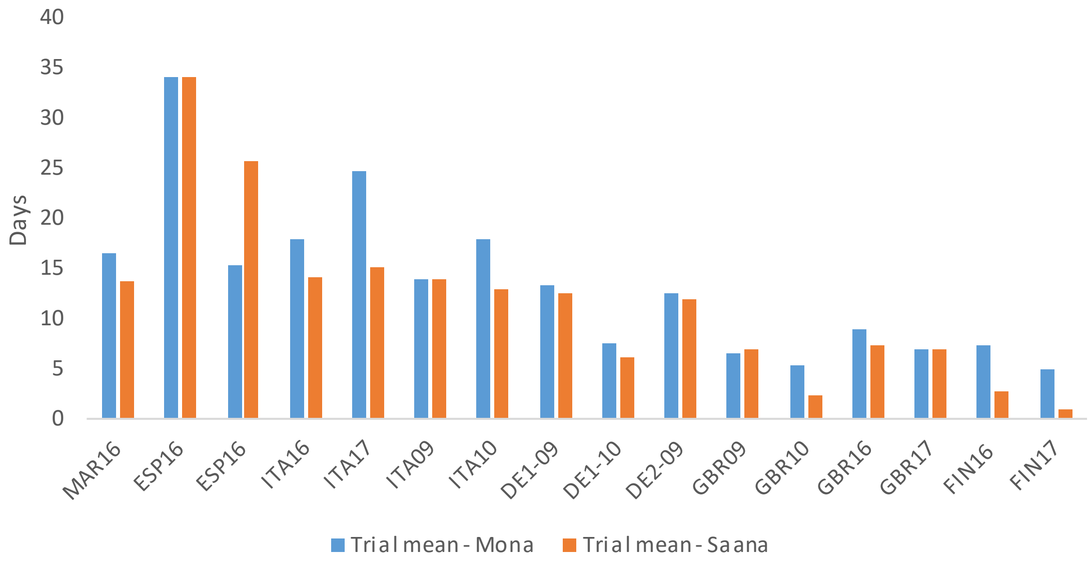

Figure S7. Differences in heading date between trial means and cultivars Mona and Saana.

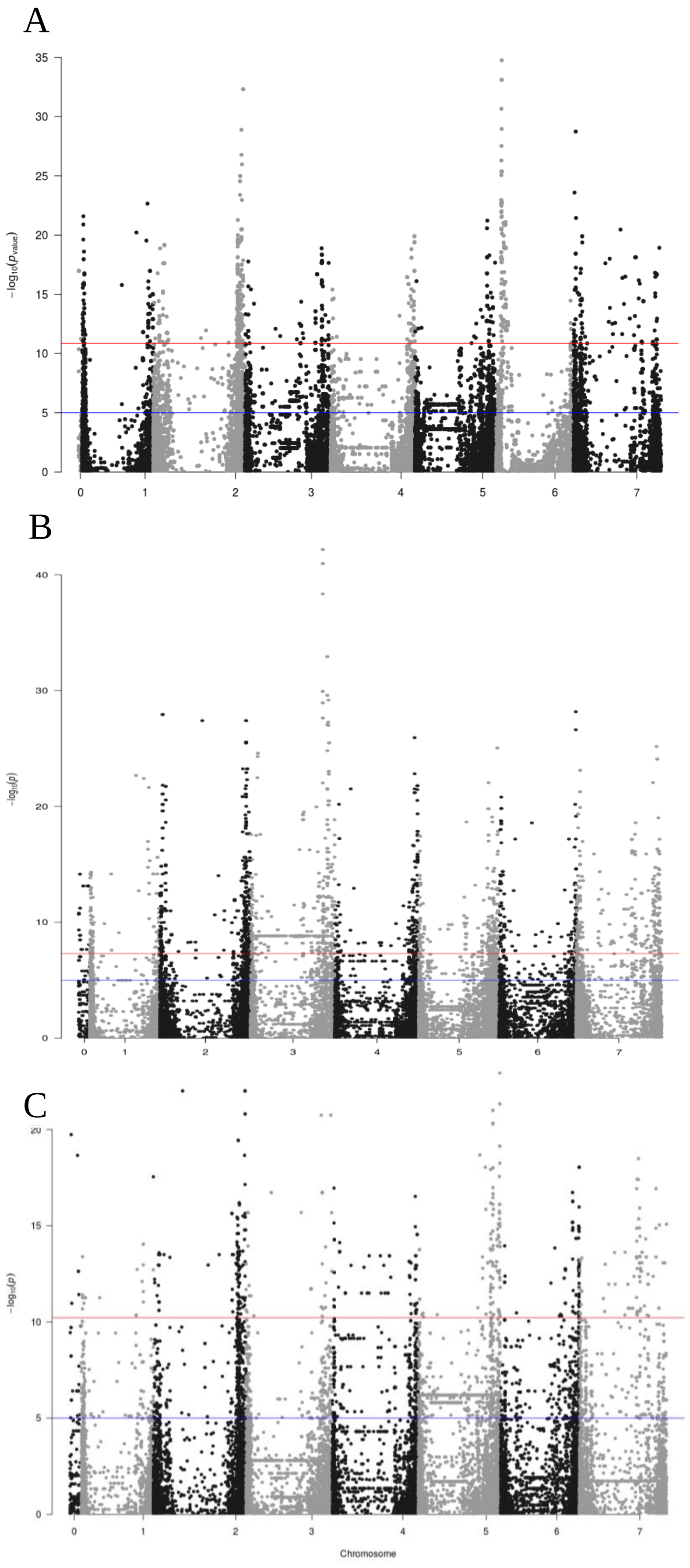

Figure S8. Manhattan plot of the meta-analysis of all trials for A) heading date, B) plant height, and C) thousand-grain weight. In blue, threshold commonly used in meta-analyses in the literature. In red, threshold calculated in this study, corresponding to the minimum -$\log_{10}$(*P*-value) resulting from 1000 permutations, specific to each trait.

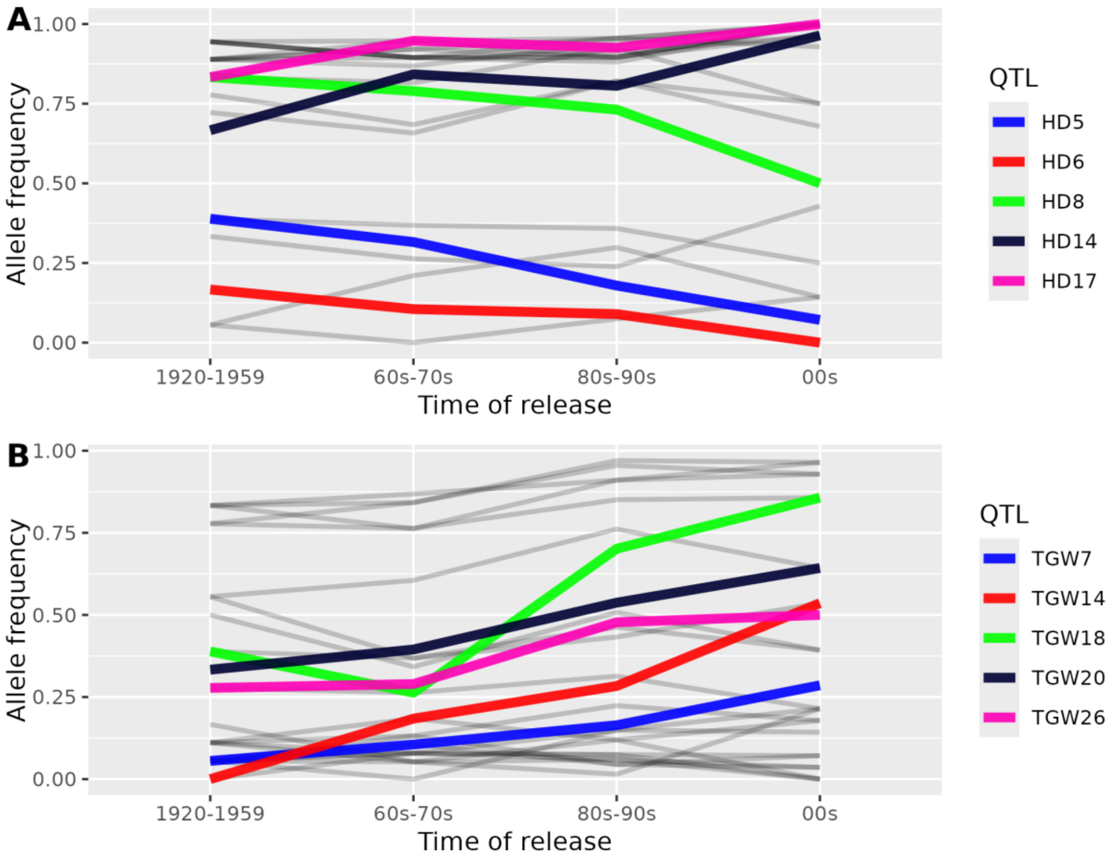

Figure S9. Changes in allele frequencies for (A) flowering time (HD) and (B) thousand-grain weight (TGW) QTLs over year of release.

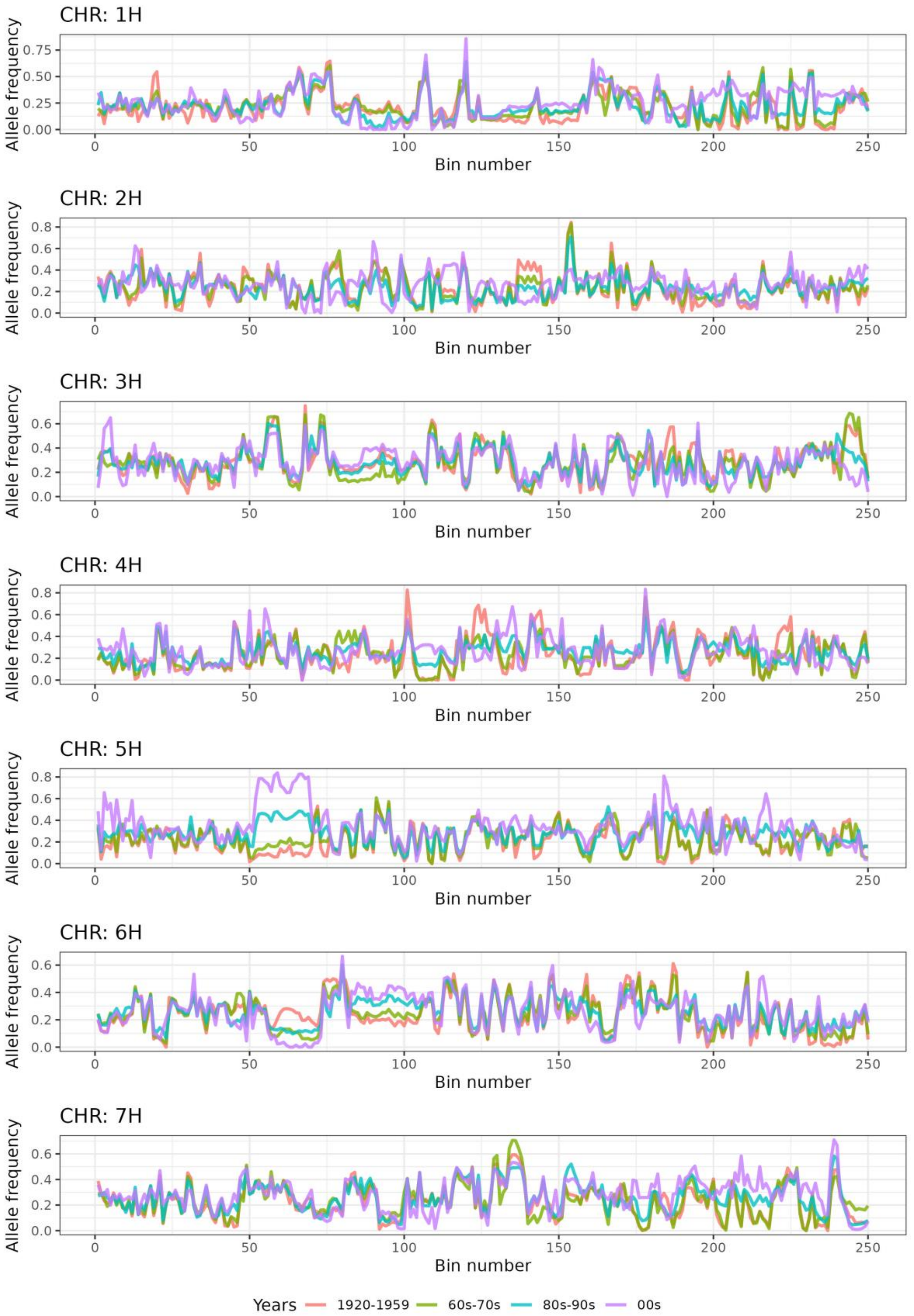

Figure S10. Genome-wide fingerprints of selection across years of selection in breeding of two-rowed spring barleys, scaled as 250 rolling windows (bins) per chromosome.

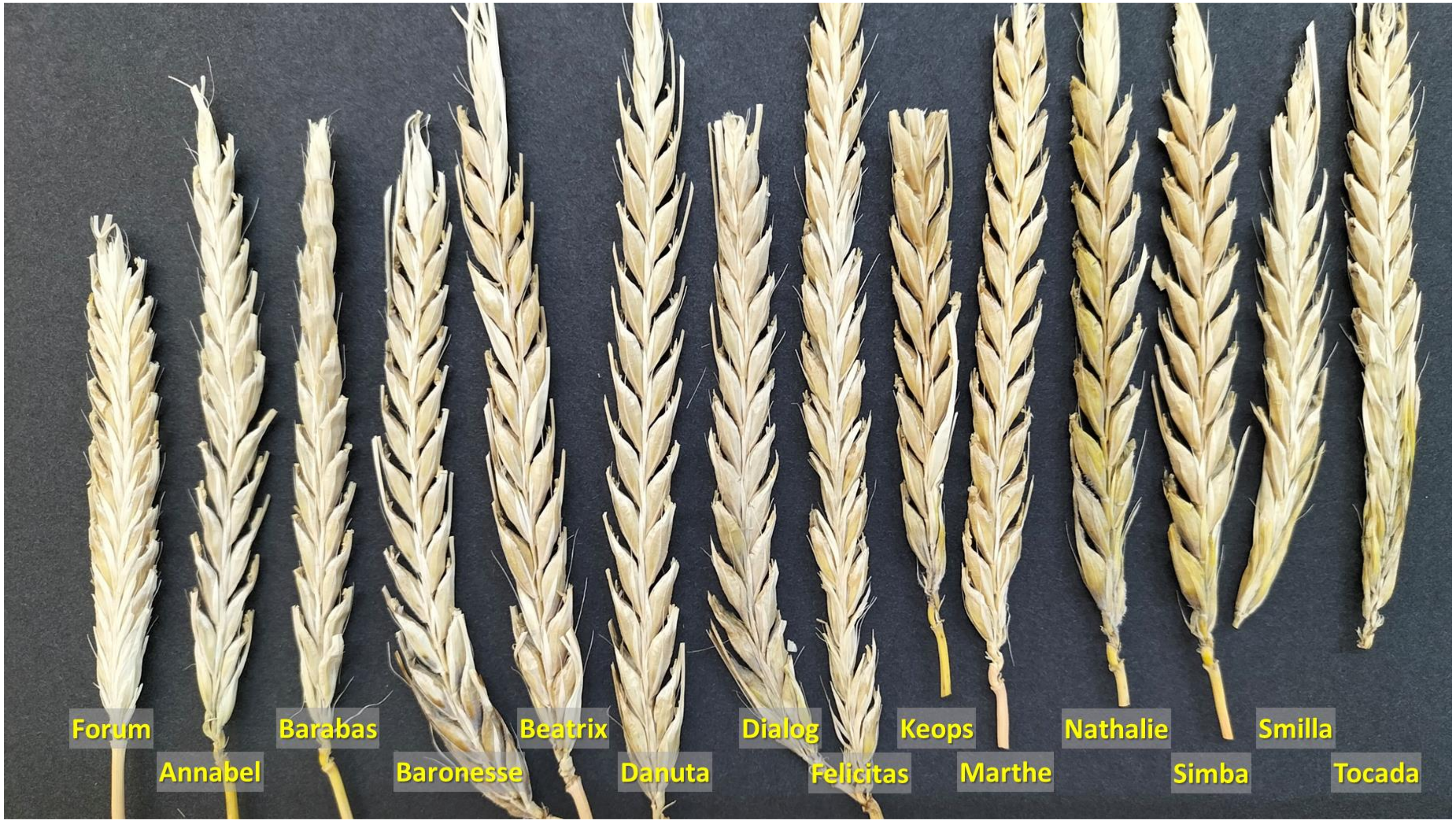

Figure S11. Picture of the spikes (awns clipped) of 14 cultivars with the favorable allele of the QTL GY2. Out of them, 13 (all but Forum) were the only carriers of the *deficiens* (G) allele of *Vrs1/HvHOX2* (Table S1), tested with a specific KASP marker. Forum showed large lateral spikelets. Felicitas and Tocada presented spikelets, but much smaller.
